# Supplementary material for: mTORC2-mediated cell-cell interactions promote BMP4-induced WNT activation and mesoderm differentiation
Source: Stem Cell Reports. 2025 Oct 16;20(11):102680. doi: 10.1016/j.stemcr.2025.102680 (PMC12790719; doi:10.1016/j.stemcr.2025.102680)
Supplement: Document S1. Figures S1–S7 and Tables S4 and S5 [file mmc1.pdf]

Stem Cell Reports, Volume *20*

Supplemental Information

mTORC2-mediated cell-cell interactions promote BMP4-induced WNT activation and mesoderm differentiation

Li Tong, Faiza Batool, Yueh-Ho Chiu, Priscilla Di Wu, Yudong Zhou, Xiaolun Ma, Santosh Atanur, and Wei Cui

SUPPLEMENTAL INFORMATION

Figure S1

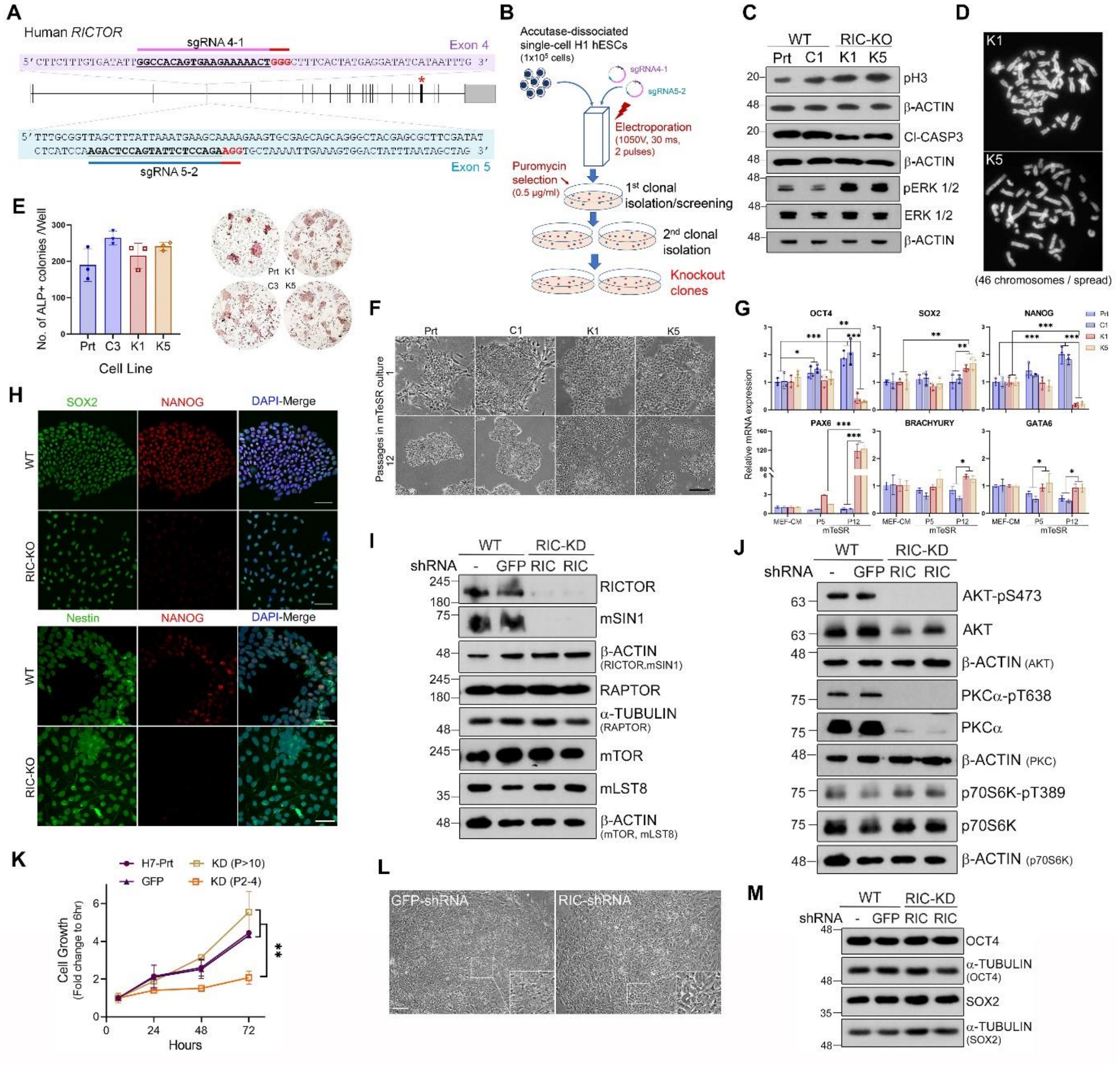

Figure S1. Generation of RICTOR-knockout and -knockdown hESCs.

(A) Diagram depicting human *RICTOR* gene, sgRNA targeting sequences and shRNA site (* in red).

(B) Schematic of generating *RICTOR*-KO clonal hESC lines.

(C) Representative immunoblots of the proliferation marker pH3 and apoptotic marker cleaved caspase 3 (Cl-CASP3) as well as ERK1/2-pThr202/Tyr204 in WT and RIC-KO hESCs (n = 3).

(D) Representative images of metaphase spreads of RIC-KO clones (n = 7 per line).

(E) Alkaline phosphatase colony formation assay. Quantitative analysis (left) shown as mean ± SD (n = 3) and representative images (right). Prt, C3 and K1, K5 represent H1 parental, control clone 3 and RIC-KO clone 1 and 5, respectively.

(F) Representative images of WT and RIC-KO hESCs in mTeSR cultures at passage 1 and 12. Scale bar = 200 μm).

(G) Comparison of mRNA expression in WT and RIC-KO hESCs under indicated culture conditions. Data are presented as mean ± SD (n = 3).

(H) Immunostaining with indicated antibodies on WT and RIC-KO hESCs cultured under mTeSR conditions for 12 passages. Scale bar = 50 μm.

(I) Immunoblots of RICTOR and mTOR complexes protein subunits in RICTOR-knockdown (RIC-KD) H7 hESCs.

(J) Immunoblots show abolished mTORC2 activities in RIC-KD H7 hESCs.

(K) Proliferation of WT and RIC-KD H7 hESCs by CCK8 assay. Indicated passage numbers of H7 RIC-KD are counted after selection. Data presented as mean ± SEM (n = 3).

(L) Representative phase-contrast images of H7 WT and RIC-KD hESCs. Scale bar = 100 μm.

(M) Immunoblots of key pluripotent transcription factors in WT and RIC-KD hESCs. All the immunoblotting experiments are repeated at least once with different culture passages (n ≥ 2).

*, ** & ***, p <0.05, 0.005 & 0.0005, respectively by two-way ANOVA.

Related to Figure 1.

Figure S2

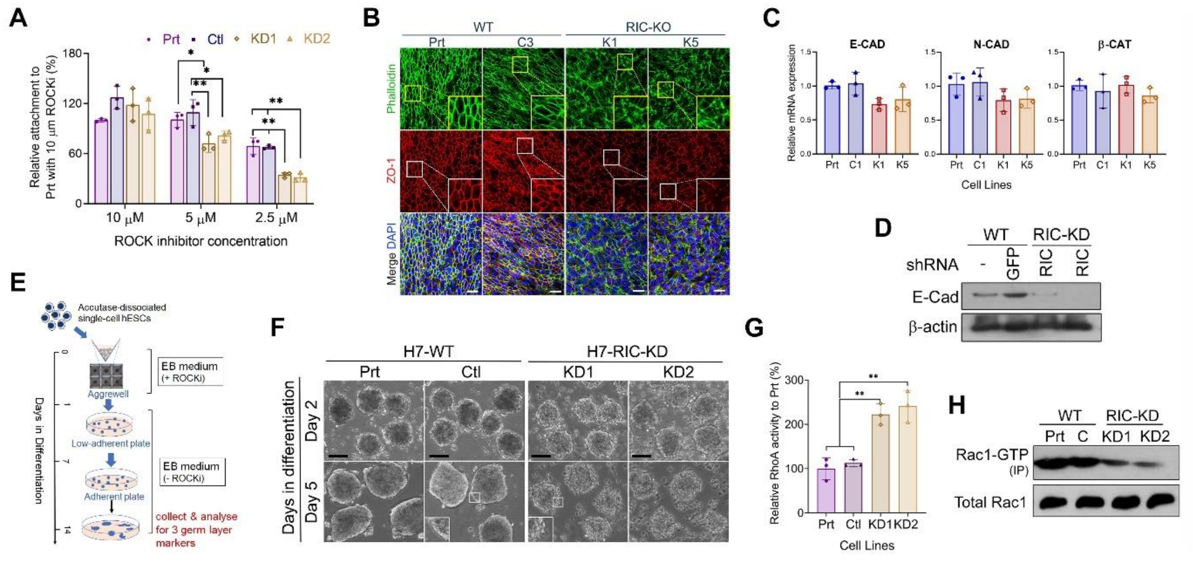

Figure S2. Effects on cell adhesion in RIC-KO and RIC-KD hESCs.

(A) Cell attachment assay in RIC-KD H7 hESCs as described in Figure 2B.

(B) Expression of ZO1 and F-ACTIN (Phalloidin) in H1 RIC-KO hESCs by immunostaining. Scale bar = 50 µm. For the enlarged inserts, the scale bar = 25 µm.

(C) RT-qPCR showing expression of adhesion complex genes, β-CATENIN (β-CAT), E- and N-CADHERIN (E-CAD and N-CAD) in H1 RIC-KO hESCs. Data are presented as mean ± SD (n = 3).

(D) E-CADHERIN expression in H7 RIC-KD and WT hESCs by immunoblotting (n = 2).

(E) Schematic illustrating the procedure of EB formation.

(F) Representative images of H7 RIC-KD EB formation in the presence of ROCKi. Insert showing blebs in RIC-KD EBs. Scale bar = 100 µm.

(G) RhoA activity in H7 WT and RIC-KD hESCs. Data are presented as mean ± SD (n = 3). ** $p < 0.005$ by one-way ANOVA.

(H) Rac1-GTP in WT and RIC-KD H7 hESCs (n = 2). KD1 & KD2 are derived from two knockdown experiments.

Related to Figure 2

Figure S3

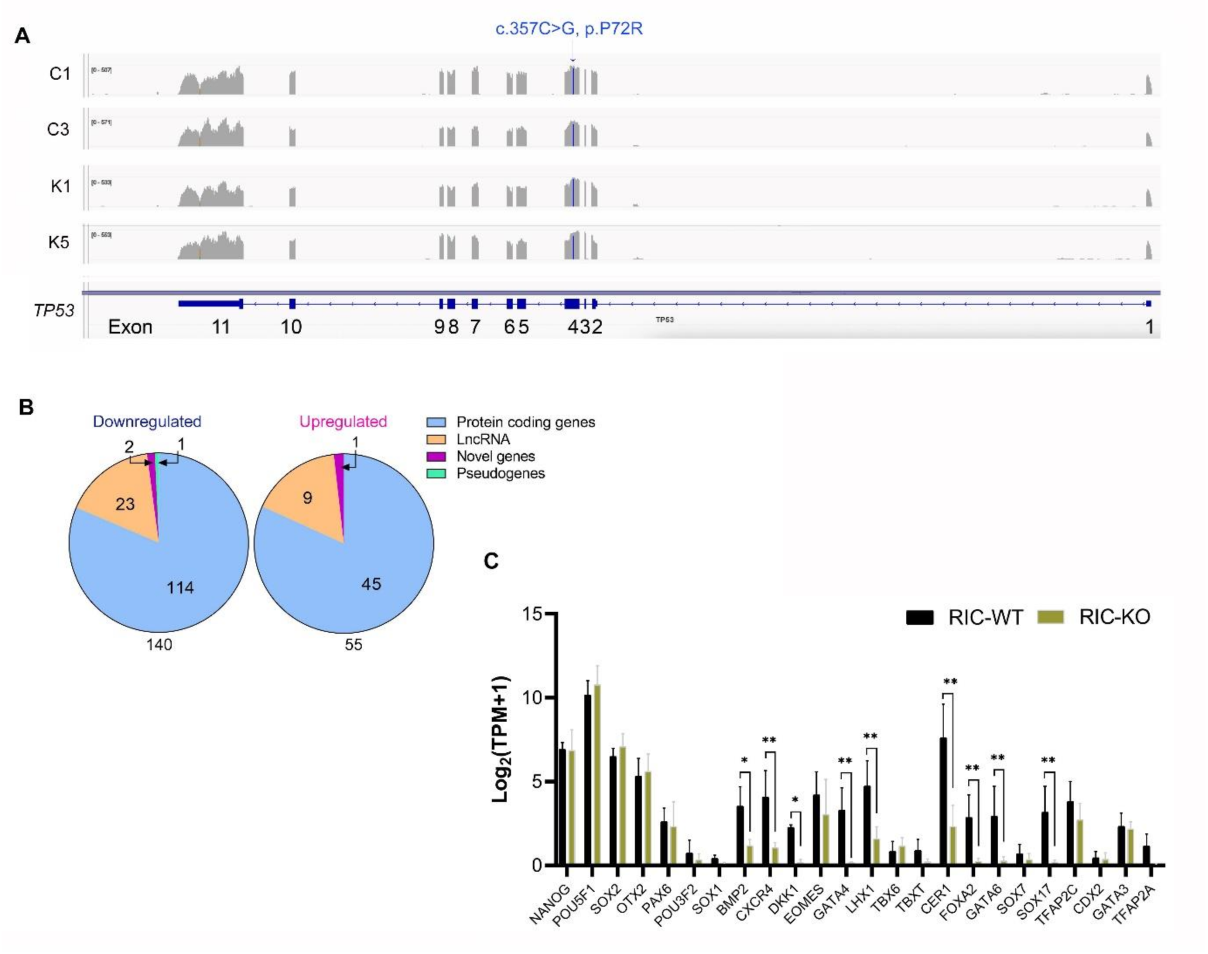

Figure S3. Differentially expressed genes in RIC-KO hESCs by RNA-seq analysis.

(A) Mapping TP53 reads onto the gene. Blue arrow points to a common polymorphism identified in exon 4 in all H1 hESC derivatives.

(B) Pie-chart showing distribution of differentially expressed genes in RIC-KO hESCs.

(C) Bar chart comparing the expression of representative lineage-associated transcription factors between WT and RIC-KO hESCs. Data are presented as mean ± SD (n = 4). * & **, $p < 0.05$ and 0.01, respectively by unpaired two tail t test.

Related to Figure 3

Figure S4

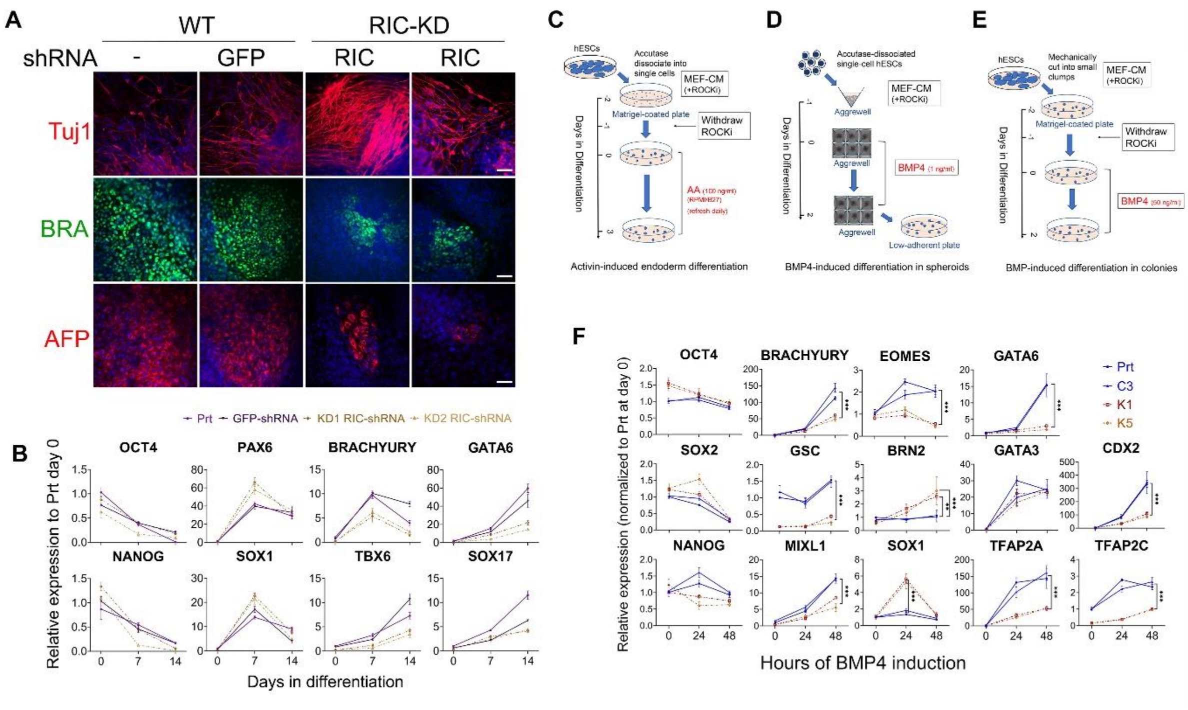

Figure S4. Reduced mesoderm/endoderm differentiation in RIC-KD and RIC-KO hESCs.

(A) Immunostaining of the three germ layer markers in WT and RIC-KD H7 hESCs after 14 days of EB differentiation (n = 2). Scale bar = 50 μm. The RIC-KD images represent two independent KD lines.

(B) Dynamic expression of indicated genes during EB differentiation in WT and RIC-KD H7 hESCs. Data are presented as mean ± SD of 6 measurements of 2 independent differentiation experiments.

(C) Schematic of Activin-induced endoderm differentiation.

(D) Schematic of BMP4-induced differentiation in spheroids.

(E) Schematic of BMP4-induced differentiation in hESC colonies.

(F) Expression of various lineage markers by RT-qPCR in BMP4-induced differentiation in WT and RIC-KO hESCs. Data are presented as mean ± SD (n = 3). ** & ***, p <0.005 and 0.0005 by two-way ANOVA.

Related to Figure 4.

Figure S5

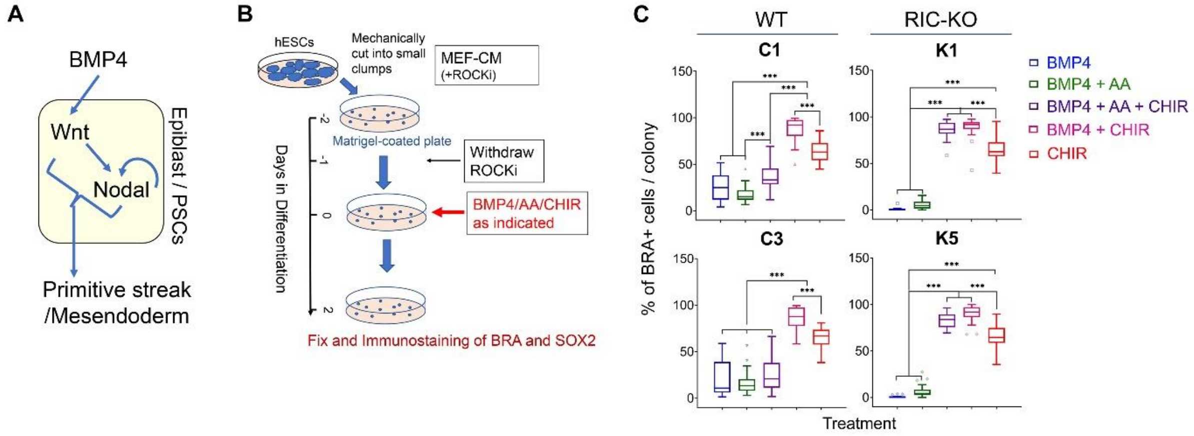

Figure S5. WNT and Activin signaling differentially affect BMP4-induced mesendoderm differentiation in WT and RIC-KO hESCs.

(A) Diagram illustrating signaling pathways involved in the regulation of gastrulation and mesendoderm differentiation.

(B) Schematic of the experimental procedure in the differentiation of hESCs with combined activation of BMP4, WNT and AA (Activin A) pathways.

(C) Comparing the effect of indicated pathways on differentiation of BRA+ cells within the same cell line. Data are presented as the Tukey box plot with number of colonies shown in Figure 4F and Figure 5 (n = 3). *, ** and ***, $p < 0.05$, 0.005 and 0.0005, respectively, by one-way ANOVA.

Related to Figure 5.

Figure S6

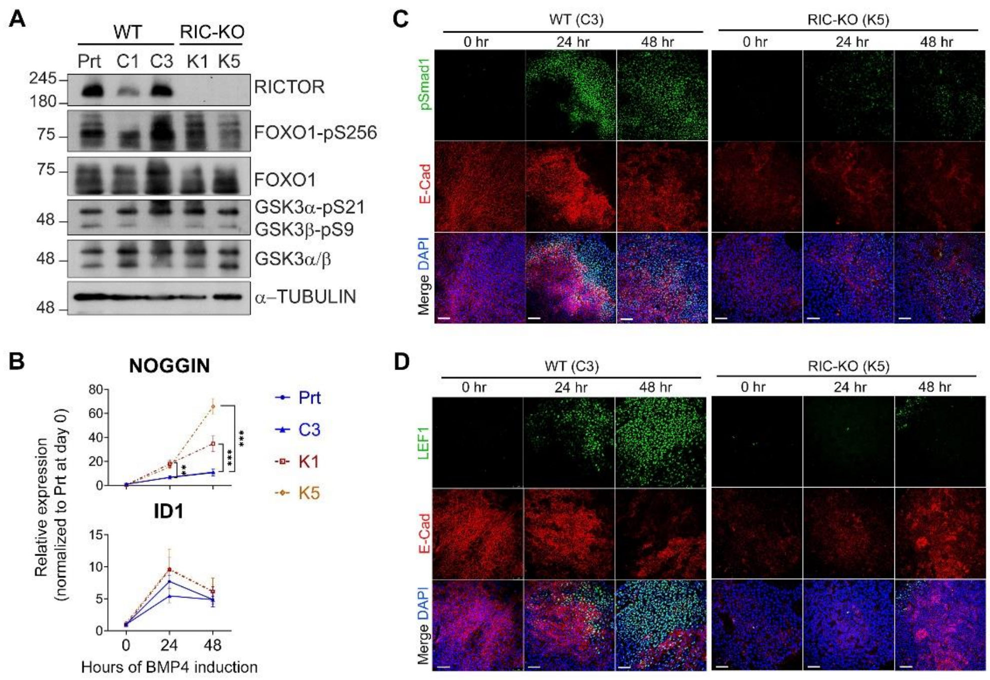

Figure S6. E-CADHERIN modulates BMP4-induced pSMAD1 signal and LEF1 expression.

(A) GSK3 and FOXO1 phosphorylation show similar patterns in WT and RIC-KO hESCs by immunoblotting (n = 2).

(B) Upregulation of BMP4 signaling target genes by RT-qPCR. Data are presented as mean ± SD(n = 3). ** and ***, p < 0.005 and 0.0005, respectively by two-way ANOVA.

(C,D) Immunostaining with indicated antibodies in C3 and K5 hESCs as described in Figure 6C,D (n = 3). Scale bar = 100 μm.

Related to Figure 6.

Figure S7

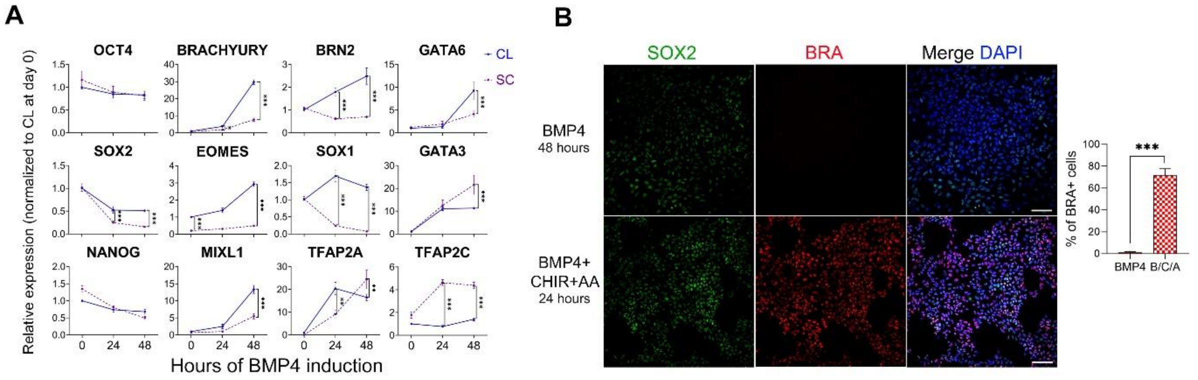

Figure S7. Cell-cell contacts affect BMP4-induced differentiation.

(A) RT-qPCR showing dynamic expression of indicated pluripotent and lineage genes during BMP4-induced differentiation in WT hESCs seeded in colonies (CL) or single cells (SC). Data are presented as mean ± SD (n = 3). ** & *** represent p < 0.005 and 0.0005 respectively by two-way ANOVA

(B) Immunostaining of BRACHYURY (BRA) and SOX2 after differentiation of SC-seeded WT hESCs by indicated treatment. Representative images are shown (left) with quantification of BRA+ cells (right) with data presented as mean ± SD of 3 independent differentiation experiments with at least three random-selected fields being counted for each experiment. ***, p <0.0005 by unpaired t-test. CHIR, CHIR99021; AA, activin A. Scale bar = 100 μm.

Related to Figure 7.

Table S4. List of RT-qPCR Primers used in this study

Gene Name	Forward primer (5'-3')	Reverse primers (5'-3')
AXIN2	GTGAGGTCCACGGAAACTGT	TGGCTGGTGCAAAGACATAG
β-ACTIN	TGTCTGGCGGCACCACCATG	AGGATGGAGCCGCCGATCCA
BRACHYURY	TGCTTCCCTGAGACCCAGTT	GATCACTTCTTTCCTTTGCATCAAG
BRN2	CCCACCCTCCATGGGAGCGT	CCAGCATGCCGTTCACCGTGA
β-CATENIN (CTNNB1)	ATTGTCCACGCTGGATTTTC	TCGAGGACGGTCGGACT
DVL2	TGAGCAACGATGACGCTGTG	GCAGGGTCAATTGGCTGGA
E-CADHERIN (CDH1)	AGCCCTTACTGCCCCCAGAG	GGGAAGATACCGGGGGACAC
EOMES	CGCCACCAAACTGAGATGAT	CACATTGTAGTGGGCAGTGG
FLK1	GGTATTGGCAGTTGGAGGAA	ACATTTGCCGCTTGGATAAC
FOXA2	GGGAGCGGTGAAGATGGA	TCATGTTGCTCACGGAGGAGTA
FZD2	CTTCTCACAGGAGGAGACGC	AAATGATAGGCCGCTCTGGG
FZD7	CGCCTCTGTTCGTCTACCTC	TCATGATGGTGCGGATACGG
GATA3	TAACATCGACGGTCAAGGCA	AGGGATCCATGAAGCAGAGG
GATA6	CAGCAAAAATACTTCCCCCA	ACTTGAGCTCGCTGTTCTCG
ID1	CAGCCAGTCGCCAAGAAT	ACAGACAGCGCACCACCT
LEF1	AATGAGAGCGAATGTCGTTGC	GCTGTCTTTCTTTCCGTGCTA
LRP6	GACTGGGTTGCACGAAATCT	CGGGGTTCCTCTAAGTCCTC
MIXL1	CCGAGTCCAGGATCCAGGTA	CTCTGACGCCGAGACTTGG
NANOG	TGATTTGTGGGCCTGAAGAAAA	GAGGCATCTCAGCAGAAGACA
N-CADHERIN (CDH2)	CAACGGGGACTGCACAGATG	TGTTTGGCCTGGCGTTCTTT
NOGGIN	GCCAGCACTATCTCCACATCCG	AGCAGCGTCTCGTTCAGATCCT
OCT4 (POU5F1)	TCGAGAACCGAGTGAGAGGC	CACACTCGGACCACATCCTTC
PAX6	TCCGTTGGAACTGATGGAGT	GTTGGTATCCGGGGACTTC
RPL22	TCGCTCACCTCCCTTTCTAA	TCACGGTGATCTTGCTCTTG
SOX1	AACACTTGAAGCCCAGATGGA	GCAGGCTGAATTCGGTTCTC
SOX2	GCCGAGTGGAAACTTTTGTCG	GCAGCGTGTACTTATCCTTCTT
SOX17	GGCGCAGCAGAATCCAGA	CCACGACTTGCCCAGCAT
TBX6	AAGTACCAACCCCGCATACA	TAGGCTGTCACGGAGATGAA
TFAP2A	GAGGTCCCGCATGTAGAA	CCGAAGAGGTTGTCCTTGT
TFAP2C	CACCTGTTGCTGCACGATCAGA	AGGAGCGACAATCTTCCAGGGA
WNT3	CGCACGACTATCCTGGAC	GAGGCGCTGTCATACTTGTC
WNT3A	TGTTGGGCCACAGTATTCCT	GGGCATGATCTCCACGTAGT
WNT5A	CCACATGCAGTACATCGGAG	CACTCTCGTAGGAGCCCTTG
WNT8A	TGTGATGGGTCAAACAATGG	TCCTTCCCCTTCTCCAAACT

Table S5. List of antibodies used in this study

Primary antibodies

Antigen	Supplier, Catalogue #	Application and Dilution
AFP	Sigma, A8452	IF: 1:1,000, IF: 1:500
AKT	Cell Signaling, 9272	IB: 1:1,000
AKT-pS473	Cell Signalling, 4060	IB: 1:1,000
α-TUBULIN	Cell Signalling, 3873	IB: 1:1,000
β-ACTIN	Proteintech, 66009	IB: 1:50,000
β-CATENIN	Cell Signalling, 8480	IB: 1:1,000, IF: 1:100
β-CATENIN	Thermo Fisher, 13-8400	IB: 1:1,000, IF: 1:100
β1-INTEGRIN	Cell Signalling, 9699	IB: 1:1,000
BRACHYURY	Cell Signaling, 81694	IB: 1:1,000, IF: 1:200
β-TUBLIN III (TUJ1)	Bio-Techne, MAB1195	IF: 1:200
E-CADHERIN	Thermo Fisher, 13-1700	IB: 1:1000, IF: 1:200
ERK1/2-pT202/Y204	Cell Signalling, 9106	IB: 1:2000
ERK1/2	Cell Signalling, 9102	IB: 1:1,000
FOXO1-pS256	Cell Signalling, 84192	IB: 1:1,000
FOXO1	Cell Signalling, 2880	IB: 1:1,000, IF: 1:100
GAPDH	Santa Cruz, sc-365062	IB: 1:5,000
GSK3α/β-pS21/9	Cell Signaling, 9331	IB: 1:1,000
GSK3α/β	Santa Cruz, sc-71190	IB: 1:500
LEF1	Cell Signaling, 2230	IB: 1:500, IF: 1:200
mLST8	Thermo Fisher, MA5-14993	IB: 1:1,000
mSIN1	Cell Signalling, 12860	IB 1:1,000
mTOR	Cell Signaling, 2983	IB: 1:500
NANOG	Cell Signaling, 4903	IB: 1:2,000, IF: 1:200
Nestin	Bio-Techne, MAB1259	IF: 1:100
OCT4	Cell Signaling, 75463	IB: 1:2,000, IF: 1:200
p70-S6K	Cell Signaling, 9202	IB: 1:1,000
p70-S6K-pT389	Cell Signaling, 9234	IB: 1:1,000
PKCα/βII-pT638/641	Cell Signaling, 9375	IB: 1:1,000
PKCα	Cell Signaling, 59754	IB: 1:1,000
PKCz-pT	Abcam, ab62372	IB: 1:2,000
PKCz	Thermo Fisher, PA517589	IB: 1:1,000
RAPTOR	Cell Signaling, 2280	IB: 1:1,000
RICTOR	Bethyl Laboratories, A300-459A	IB: 1:2,000
SMAD1/5/9-pS463/465/467	Cell Signaling, 13820	IB: 1:1,000, IF: 1:800
SMAD1	Cell Signaling, 6944	IB: 1:1,000
SMAD2-pS465/467	Cell Signaling, 3108, 18338	IB: 1:1,000
SMAD2/3	Cell Signaling, 8685	IB: 1:1,000
SOX2	Bio-Techne, AF2018	IB: 1:2,000, IF: 1:300
SSEA1	Thermo Fisher, MA1-022	Flow: 1:100
SSEA4	Thermo Fisher, MA1-021	Flow: 1:50-1:100
Tra-1-81	Thermo Fisher, MA1-024	Flow: 1:100
ZO1	Thermo Fisher, 33-9100	IB: 1:2,000, IF: 1:200

Secondary antibodies

Goat IgG-HRP	Santa Cruz, sc-2354	IB: 1:10,000

Mouse IgG (H+L)-HRP	Thermo Fisher, G-21040	IB: 1:5,000-1:20,000
Rabbit IgG (H+L)-HRP	Thermo Fisher, 31462	IB: 1:2,000-1:10,000
Mouse IgG3 Isotype Control	Thermo Fisher, MG300	Flow: 1:100
Mouse IgM Isotype Control	Thermo Fisher, 02-6800	Flow: 1:100
Goat IgG-Alexa Fluor™ 488	Thermo Fisher, A-11055	IF: 1:500
Mouse IgG-Alexa Fluor™ 488	Thermo Fisher, A-11001	IF: 1:500
Mouse IgG-Alexa Fluor™ 568	Thermo Fisher, A-11004	IF: 1:500
Rabbit IgG-Alexa Fluor™ 488	Thermo Fisher, A-11008	IF: 1:500
Rabbit IgG-Alexa Fluor™ 568	Thermo Fisher, A-11011	IF: 1:500
