# Supplementary material for: mTORC2-mediated cell-cell interactions promote BMP4-induced WNT activation and mesoderm differentiation
Source: Stem Cell Reports. 2025 Oct 16;20(11):102680. doi: 10.1016/j.stemcr.2025.102680 (PMC12790719; doi:10.1016/j.stemcr.2025.102680)
Supplement: Document S2. Article plus supplemental information [file mmc5.pdf]

Stem Cell Reports
Article
OPEN ACCESS

mTORC2-mediated cell-cell interactions promote BMP4-induced WNT activation and mesoderm differentiation

Li Tong,[1,3] Faiza Batool,[1,3] Yueh-Ho Chiu,[1,3] Priscilla Di Wu,[1] Yudong Zhou,[1] Xiaolun Ma,[1] Santosh Atanur,[2] and Wei Cui[1,4,*]

[1]Institute of Reproductive and Developmental Biology, Department of Metabolism, Digestion and Reproduction, Faculty of Medicine, Imperial College London, London W12 0NN, UK
[2]Section of Genetics and Genomics, Department of Metabolism, Digestion and Reproduction, Faculty of Medicine, Imperial College London, London W12 0NN, UK
[3]These authors contributed equally
[4]Lead contact
*Correspondence: wei.cui@imperial.ac.uk
https://doi.org/10.1016/j.stemcr.2025.102680

SUMMARY

The mechanistic target of rapamycin complex 2 (mTORC2) is essential for embryonic development, but its underlying molecular mechanisms remain unclear. Here, we show that disruption of mTORC2 in human embryonic stem cells (hESCs) considerably alters the Rho/Rac signaling dynamics and reduces E-cadherin expression and cell adhesion. Despite this, mTORC2-deficient hESCs maintain self-renewal and expression of pluripotent markers when cultured in mouse embryonic fibroblast conditioned medium (MEF-CM) supplemented with bFGF. However, these hESCs exhibit significantly impaired mesoderm and endoderm differentiation in response to BMP4 and Activin treatment, respectively, possibly due to reduced WNT activation mediated by cell-cell interactions. Direct activation of the WNT pathway using a GSK3 inhibitor restores mesendoderm differentiation in mTORC2-deficient hESCs. Our study uncovers a novel mechanism by which mTORC2 regulates cell fate determination and highlights a critical link between the intercellular adhesion and the activation of canonical WNT genes.

INTRODUCTION

The mechanistic/mammalian target of rapamycin (mTOR) is a serine/threonine protein kinase that is evolutionarily conserved in all eukaryotes. It functions through two distinct multiprotein complexes, mTORC1 and mTORC2, both of which form homodimers (Aylett et al., 2016; Scaiola et al., 2020). Although both complexes contain mTOR as the catalytic subunits and mammalian lethal with SEC13 protein 8 (mLST8), each complex also contains specific components. RAPTOR (regulatory-associated protein of mTOR), which is specific to mTORC1, is replaced by RICTOR (rapamycin-insensitive companion of mTOR) and mSIN1 (mammalian stress-activated map kinase-interacting protein 1) in mTORC2. These complex-specific components dictate their distinct regulatory mechanisms, substrates, and functions (Figure 1A) (Battaglioni et al., 2022). mTOR signaling plays an important role in cell proliferation, cytoskeleton remodeling, and cell metabolism; hence, it is essential for embryonic development and tissue homeostasis (Liu and Sabatini 2020). Over the past decades, significant advances have been made in understanding of mTORC1 (Battaglioni et al., 2022; Kim and Guan 2019), while the functions and underlying molecular mechanisms of mTORC2 remain less well defined due to its intricate crosstalk with other pathways, interwoven regulatory machinery, and the absence of mTORC2-specific inhibitors.

Loss-of-function studies in mice demonstrate that mTORC2 is vital for embryonic development. Disruption of either *Rictor* or *Mapkap1*, gene encoding mSin1, leads to embryonic lethality at E10–11 (Guertin et al., 2006; Shiota et al., 2006), which occurs later than the death of Raptor-null embryos at E6.5, suggesting that mTORC1 and mTORC2 have diverse functions in embryonic development. Intriguingly, tissue-specific knockout of mTORC2 in mice, such as in endothelial cells, generally results in mild phenotypes without overt developmental defects, unless the disruption occurs around gastrulation; in such cases, the phenotype closely resembles that of a germline knockout (Aimi et al., 2015; Bentzinger et al., 2008; Cybulski et al., 2009; Dragert et al., 2015; Godel et al., 2011; Hagiwara et al., 2012; Lamming et al., 2012; Zhao et al., 2014). These imply that mTORC2 may have an important function during early embryonic development, particularly around gastrulation, yet the underlying molecular mechanisms are largely unknown. Additionally, the role of mTOR signaling, especially mTORC2, in pluripotent stem cells (PSCs) also remains elusive (Bulut-Karslioglu et al., 2016; Zhou et al., 2009).

To explore the role of mTORC2 in embryonic development and PSCs, we disrupted the expression of *RICTOR* in human embryonic stem cells (hESCs) and characterized the resulting cells for their self-renewal and cell fate determination. *RICTOR*-knockout (RIC-KO) hESCs exhibit a considerable reduction in cell adhesion and interactions with a decreased propensity for mesoderm and endoderm

Published by Elsevier Inc. on behalf of International Society for Stem Cell Research.
This is an open access article under the CC BY license (http://creativecommons.org/licenses/by/4.0/).

differentiation. Further studies revealed that mTORC2-mediated cell interactions are crucial for mesoderm and endoderm differentiation through modulation of WNT signaling.

RESULTS

Eradicating mTORC2 signaling shows no effect on self-renewal of hESCs

The *RICTOR* gene was disrupted in H1 hESCs using CRISPR-Cas9, and two RIC-KO clones (K1 and K5) were expanded in mouse embryonic fibroblast conditioned medium (MEF-CM) (Figures S1A and S1B). Elimination of *RICTOR* dramatically reduced mSIN1 protein levels without affecting other core subunits of the mTOR complexes as reported before (Yang et al., 2006) (Figure 1B). As expected, mTORC2 activity was abolished, while mTORC1 signaling remained (Figure 1C). During initial clonal expansion, RIC-KO hESCs showed a transient reduction in cell growth, which gradually diminished. When clonal lines were established, RIC-KO hESCs revealed faster growth than wild-type (WT) controls with increased mitogen-activated protein kinase (MAPK)-ERK pathway activity (Figure 1D; Figure S1C). Interestingly, upon differentiation into fibroblasts, these RIC-KO cells grew slower than WT cells (Figure 1D), which is in line with fibroblasts derived from *Rictor*-null mouse embryos (Guertin et al., 2006; Shiota et al., 2006). Both RIC-KO hESC lines maintained normal chromosomes and formed alkaline phosphatase-positive colonies similar to WT controls (Figures S1D and S1E; Figure 1E). Expression of key pluripotent transcription factors and hESC-associated cell surface antigens showed no clear change (Figures 1F–1I). However, when cultured in chemically defined mTeSR medium, the RIC-KO hESCs gradually lost NANOG expression although SOX2 expression was still high (Figures S1F–S1H) as previously reported (Chu et al., 2022). These results suggest that culture conditions have crucial effects on RIC-KO hESCs.

To circumvent off-target effects of single-guide RNAs (sgRNAs) and cell line-dependent bias, *RICTOR* expression was also knocked down in H7 hESCs using short hairpin RNA (shRNA) (RIC-KD) (Figures S1I and S1J). These cells exhibited similar growth dynamics, morphology, and marker expressions to H1 RIC-KO hESCs (Figures S1K–S1M). Together, these data demonstrate that disrupting RICTOR in hESCs eliminates mTORC2 activity without affecting mTORC1 signaling and that mTORC2 is not essential for the maintenance of hESC self-renewal in the MEF-CM culture conditions.

mTORC2 deficiency in hESCs significantly reduces cell adhesion and alters RhoA/Rac signaling

In our routine culture, hESCs were propagated by collagenase-assisted mechanical splitting, and small cell clumps were plated at a 1:3 ratio onto Matrigel-coated plates (Noisa et al., 2011). Although *RICTOR* disruption showed no apparent effect on hESC self-renewal, both H1 RIC-KO and H7 RIC-KD (together called RICTOR-deficient, RIC-DF) hESCs exhibited noticeably reduced attachments to culture plates, but this defect was visibly ameliorated by the addition of the ROCK inhibitor Y-27632 (ROCKi) (Figures 2A and 2B; Figure S2A). Furthermore, intercellular junction proteins, including E-cadherin (E-CAD) and ZO1, showed lower levels in RIC-DF hESCs without significant changes on the transcript levels (Figures 2C and 2D; Figures S2B–S2D). β-CATENIN (β-CAT) protein also displayed reduced localization at the cell membrane. These findings are consistent with the observation that RIC-DF hESC colonies were less compact than WT controls (Figure 1E; Figure S1L), suggesting reduced cell interactions in RIC-DF hESCs.

The impeded cell interactions in RIC-DF hESCs became more evident during embryoid body (EB) formation. In contrast to WT hESCs that require ROCKi only on the first day (Figure S2E), RIC-DF hESCs required continuous ROCKi treatment throughout EB formation; without it, cell aggregates disintegrated (Figure 2E). Notably, the disintegrated cells remained viable, as indicated by negative trypan blue staining. Even with ROCKi, RIC-DF EBs showed more surface blebbing (Figure 2E; Figure S2F). These results reveal that RICTOR/mTORC2-deficiency significantly hinders cell-ECM (extracellular matrix) and cell-cell interactions.

Since ROCKi partially rescued the attachment defects of RIC-DF hESCs, RhoA-ROCK signaling was thought to be higher in these cells. Indeed, RhoA activity was significantly increased in RIC-DF hESCs (Figure 2F; Figure S2G), while the levels of Rac1-GTP were reduced (Figure 2G; Figure S2H). Phalloidin staining further showed an altered pattern of F-ACTIN organization in RIC-KO hESCs, implying a change in cytoskeleton (Figure S2B). RhoA and Rac1 are well known to function antagonistically and coordinately regulate cell adhesion, cytoskeleton, and movement (Burridge and Wennerberg 2004; Martin et al., 2016; Yang et al., 2006). Our results suggest that mTORC2 plays an important role in maintaining the balance of RhoA-ROCK/Rac1 signaling in hESCs and that disruption of mTORC2 considerably upregulates RhoA-ROCK, contributing to impaired cell adhesion and cytoskeletal disorganization.

Disrupting mTORC2 in hESCs downregulates genes involved in mesoderm differentiation and WNT signaling

To assess the impact of mTORC2 disruption in hESCs, RNA sequencing (RNA-seq) analysis was performed on WT (C1 and C3) and RIC-KO (K1 and K5) hESCs. The vast majority

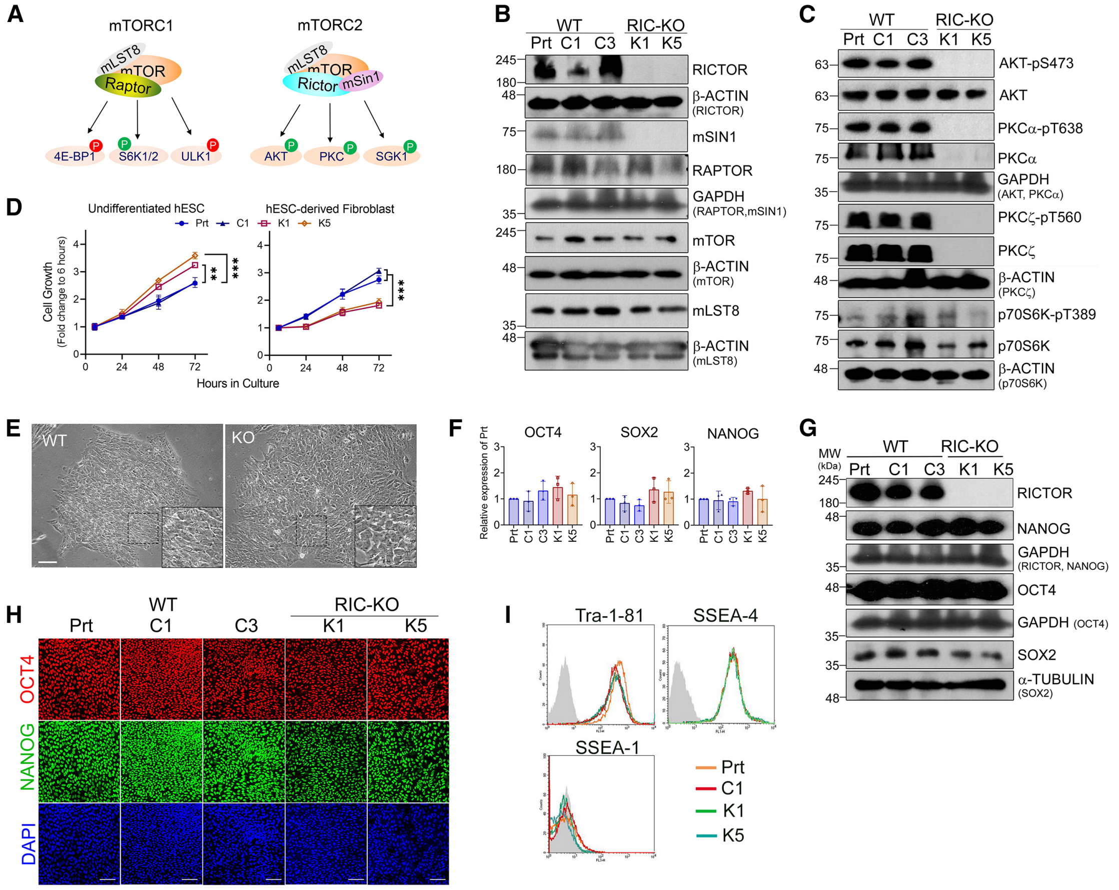

Figure 1. RIC-KO hESCs retain the self-renewal and pluripotent marker expression

(A) Schematics illustrate mTOR complexes their promoting and inhibitory substrates, green and red P, respectively.

(B and C) The expression of mTORC1 and mTORC2 key subunits (B) and the phosphorylation on their targets (C) ($n > 2$) by immunoblotting. Prt, parental H1 hESCs; C1 and C3, WT controls; K1 and K5, RIC-KO clones.

(D) Proliferation of hESC lines and their differentiated fibroblasts by CCK8 assay. Data are presented as mean ± SEM ($n = 3$). ** and ***$p < 0.005$ and 0.0005, respectively, by two-way ANOVA.

(E) Phase-contrast images of WT and RIC-KO hESCs. Scale bars, 50 μm.

(F) Expression of key pluripotent transcription factors in hESCs by reverse-transcription qPCR (RT-qPCR). Data are presented as mean ± SD ($n = 3$).

(G and H) Protein expression of key pluripotent transcription factors in hESCs by immunoblotting (G) and immunostaining (H) ($n > 2$). Scale bars, 25 μm.

(I) Expression of hESC-associated cell surface antigens in hESCs by flow cytometry ($n = 2$).

See also Figure S1.

of genes showed no significant change in expression; no *TP53* mutation was found in all the cell lines (Figure S3A). Only 0.6% (195 out of 32,673 genes) were differentially expressed (>2-fold change, adjusted $p < 0.05$), including 140 downregulated and 55 upregulated genes (Figures 3A and 3B; Figure S3B; also see Tables S1 and S2). Notably, several downregulated genes encode transcription factors vital for lineage specification, particularly mesoderm and endoderm, including *GATA4*, *FOXA2*, *SOX17*, *and SP5* (Mukherjee et al., 2020; Rojas et al., 2005; Weidinger et al., 2005). In contrast, genes associated with pluripotency or neural differentiation were expressed at similar levels (Figure 3B; Figure S3C). Since mTORC2 primarily regulates protein phosphorylation, it is not surprising that genes directly involved in cell adhesion, such as E-CAD, were not transcriptionally altered (Figure S2C). Although no statistically significant results were obtained on the upregulated genes by Gene Ontology (GO) enrichment analysis, the analysis of the downregulated genes showed significant association with embryonic development, embryo patterning, and muscle formation, as well as regulation of gene expression and growth

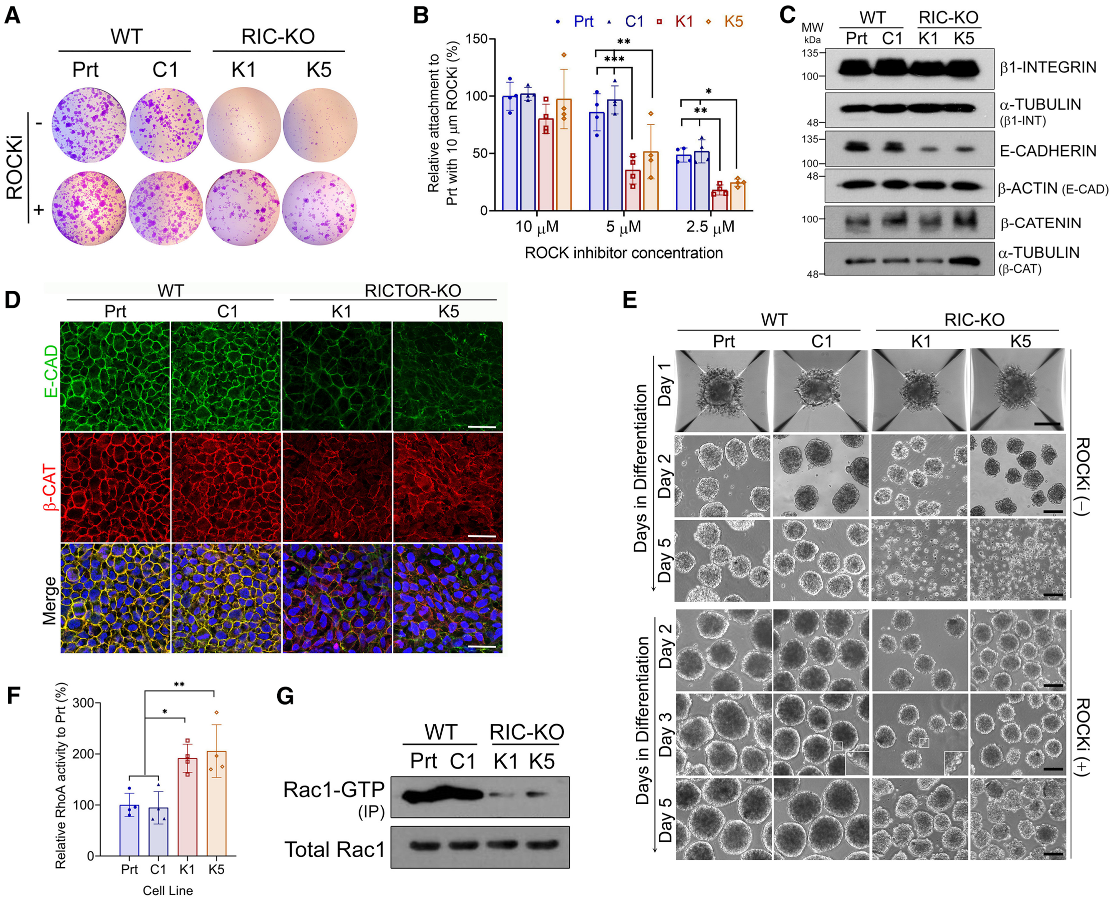

Figure 2. RIC-KO hESCs exhibit reduced cell adhesion

(A) Images of violet staining of hESC colonies 24 h after plating in the presence or absence of the ROCK inhibitor (ROCKi) (n = 3).
(B) Cell attachment assay with indicated concentration of ROCKi. Data are presented as mean ± SD (n = 4). *, **, and ***p < 0.05, 0.01, and 0.0005 by two-way ANOVA.
(C) Expression of proteins associated with cell adhesion by immunoblotting ($n \geq 2$).
(D) Immunostaining of WT and RIC-KO hESCs with indicated antibodies. Scale bars, 30 μm.
(E) Phase-contrast images of EB formation (n = 2) in hESCs in the absence or presence of ROCKi (n = 2). Inserts on day 3 showing blebs in RIC-KO EBs. Scale bars, 100 μm.
(F) RhoA activity in hESCs. Data are presented as mean ± SD (n = 4). * and **p < 0.05 and 0.005, respectively, by one-way ANOVA.
(G) Rac1-GTP pull-down assay in hESCs (n = 2).
See also Figure S2.

factor/cytokine activity (Figures 3C and 3D; Table S3). Kyoto Encyclopedia of Genes and Genomes (KEGG) pathway analysis further linked these genes to WNT signaling (Figure 3E). These findings suggest that mTORC2 may have a role in hESC differentiation by modulating the expression of lineage-specific genes, possibly through regulation of WNT signaling.

mTORC2 deficiency hinders mesoderm and endoderm differentiation of hESCs

To evaluate the differentiation potential of RIC-DF hESCs, EB-mediated differentiation was performed in the presence of ROCKi (Figure S2E). Although RIC-DF EBs contained derivatives of all three germ layers, the proportions of mesodermal and endodermal cells were much less than WT controls (Figure 4A; Figure S4A). This was corroborated by mRNA expression analysis, showing significantly lower levels of mesoderm (BRACHYURY, TBX6, and FLK1) and endoderm (FOXA2, GATA6, and SOX17) markers in RIC-DF EBs (Figure 4B; Figure S4B).

To further assess endodermal differentiation, RIC-KO hESCs were also subjected to Activin A (AA)-induced differentiation (Figure S4C), a process previously shown to be negatively regulated by phosphatidylinositol

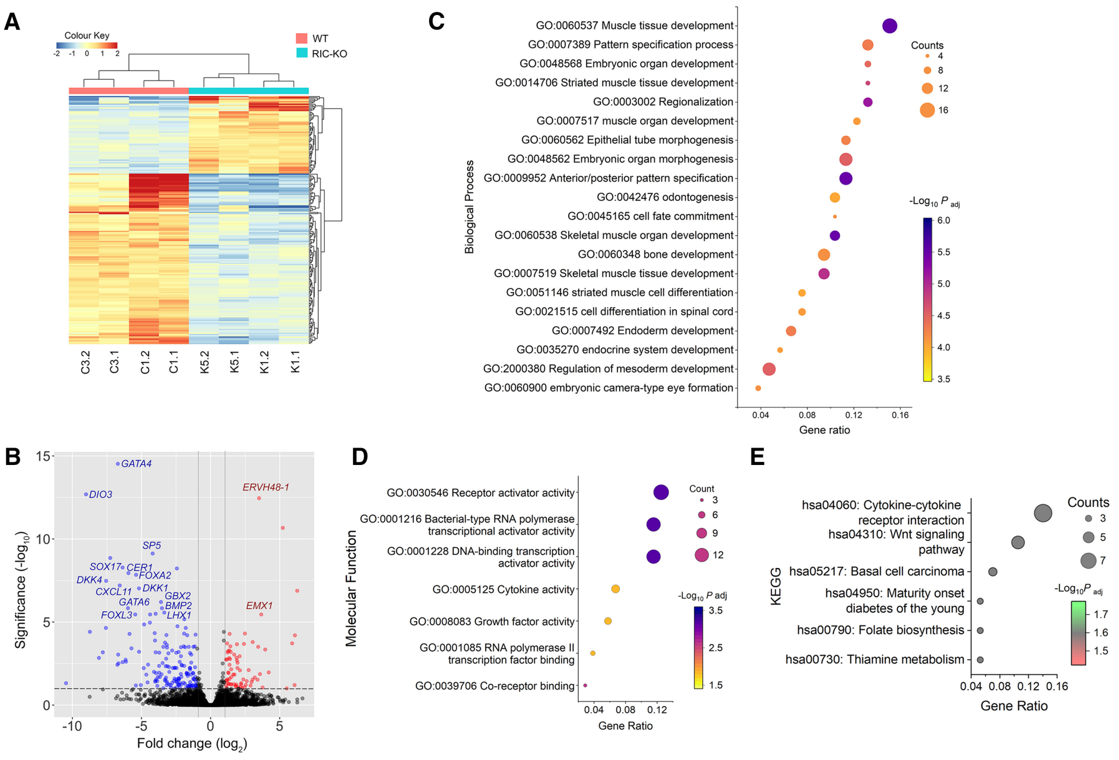

Figure 3. Altered expression of developmental-associated genes in RIC-KO hESCs by RNA-seq
(A) Heatmap and hierarchical clustering showing differentially expressed genes in WT and RIC-KO hESCs ($n = 2$).
(B) Volcano plots of differentially expressed genes (adjusted p value [*Padj*] < 0.05; log2 [fold change] > 1) in RIC-KO hESCs. Blue and red dots represent downregulated and upregulated genes, respectively.
(C and D) Gene ontology enrichment of downregulated genes in biological process (C) and molecular function (D) (*Padj* < 0.05).
(E) KEGG pathway enrichment (*Padj* < 0.05) of downregulated genes.
See also Figure S3.

3-kinase/mTORC2 signaling (Yu et al., 2015). Unexpectedly, RIC-KO hESCs exhibited significantly impaired endoderm differentiation despite the prolonged AA-induced pSmad2/3 signal duration (Figures 4C and 4D), consistent with prior findings (Yu et al., 2015). Notably, expression of BRACHURY, a mesendoderm marker, was also reduced in RIC-KO cells (Figures 4C and 4D), indicating that impaired mesendoderm might underlie the observed deficiency in endoderm differentiation. This aligns with the role of mesendoderm as a bipotent precursor for both mesoderm and endoderm, resembling cells of the primitive streak in gastrulating embryos (Rodaway and Patient 2001).

To further validate the role of mTORC2 in mesendoderm differentiation, we adapted a BMP4-induced differentiation protocol in 2D colony and 3D spheroid cultures (Figures S4D and S4E) (Bernardo et al., 2011; Simunovic et al., 2019). After 48 h of BMP4 induction, WT hESCs exhibited symmetry breaking, characterized by the emergence of BRACHYURY-positive (BRA^+) cells one side of a colony or spheroid and SOX2-positive cells on the opposite side (Figures 4E and 4F), consistent with previous results (Simunovic et al., 2019). In contrast, RIC-KO cultures hardly showed BRA^+ cells, indicating a failure to initiate mesendoderm differentiation. Correspondingly, expression of mesoderm/endoderm genes was significantly reduced in RIC-KO cells (Figure S4F). Taken together, these results demonstrate that ablation of mTORC2 in hESCs considerably impedes their mesendoderm differentiation, implying that mTORC2 has a regulatory role in early lineage specification.

Activation of WNT signaling recovers mesendoderm differentiation in RIC-KO hESCs

Next, to investigate the underlying mechanisms by which mTORC2 regulates mesendoderm differentiation, we focused on BMP, Wnt, and Nodal signaling pathways, which are known to orchestrate gastrulation in mouse embryos (Nowotschin and Hadjantonakis 2020). In this signaling cascade, BMP4 secreted from extraembryonic tissues activates Wnt signaling in epiblast, which in turn enhances Nodal, collectively promoting the formation of the primitive streak/mesendoderm (Figure S5A). Similar

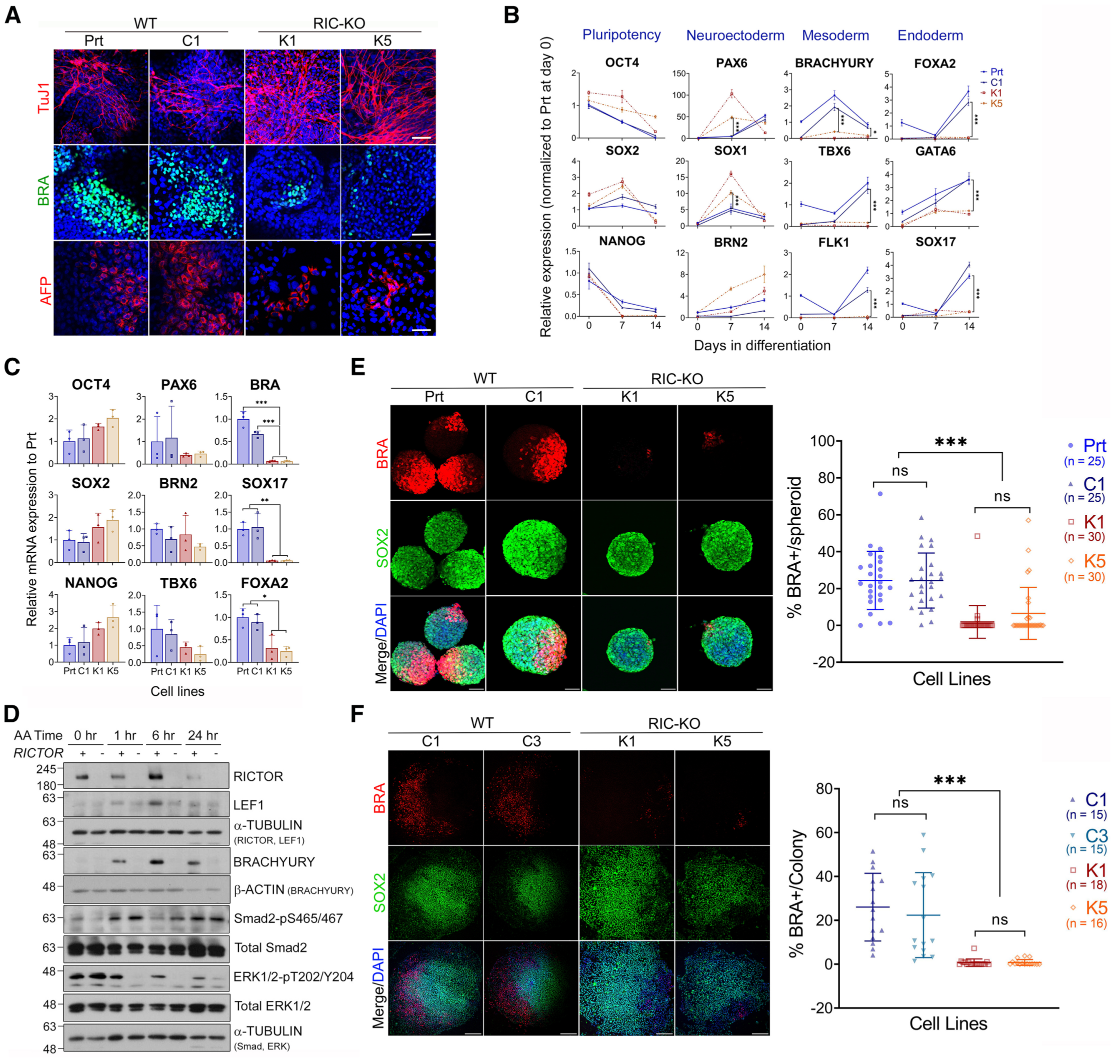

Figure 4. mTORC2 deficiency impedes mesoderm and endoderm differentiation
(A) Immunostaining of the three germ layer markers after 14-day differentiation via EB (n = 2). Scale bars, 50 μm.
(B) Dynamic mRNA expression during EB differentiation by RT-qPCR. Data are presented as mean ± SD of 6 measurements of 2 independent differentiation experiments.
(C) Expression of indicated pluripotent and lineage genes after 72 h of Activin-induced endoderm differentiation. Data are presented as mean ± SD (n = 3).
(D) Expression of indicated proteins during Activin-induced endoderm differentiation by immunoblotting in WT and RIC-KO hESCs.
(E) Max intensive projection images of immunostaining with Brachyury (BRA) and SOX2 antibodies (left; scale bars, 75 μm) and quantification of % BRA$^+$ cells/spheroid (right).
(F) Images of immunostaining with Brachyury (BRA) and SOX2 antibodies (left; scale bars, 250 μm) and quantitative analysis (right). The quantification in (E) and (F) is presented as mean ± SD with each symbol representing a spheroid/colony with the total number analyzed of each line from 3 independent experiments indicated in the bracket. *, **, and ***p < 0.05, 0.005, and 0.0005 by one-way ANOVA. See also Figure S4.

regulatory mechanisms have been observed in non-human primates, indicating their conservation between species (Cui et al., 2022). To test whether mTORC2-deficient hESCs are competent to respond to these signals, both WT and RIC-KO hESCs were treated with BMP4, CHIR99021 (CHIR; a GSK3 inhibitor to activate WNT signaling), and AA (a NODAL pathway agonist) (Figure S5B). This combined treatment substantially increased the proportion of BRA$^+$ cells in RIC-KO cultures, even exceeding the proportion observed in WT controls (Figure 5A). These results

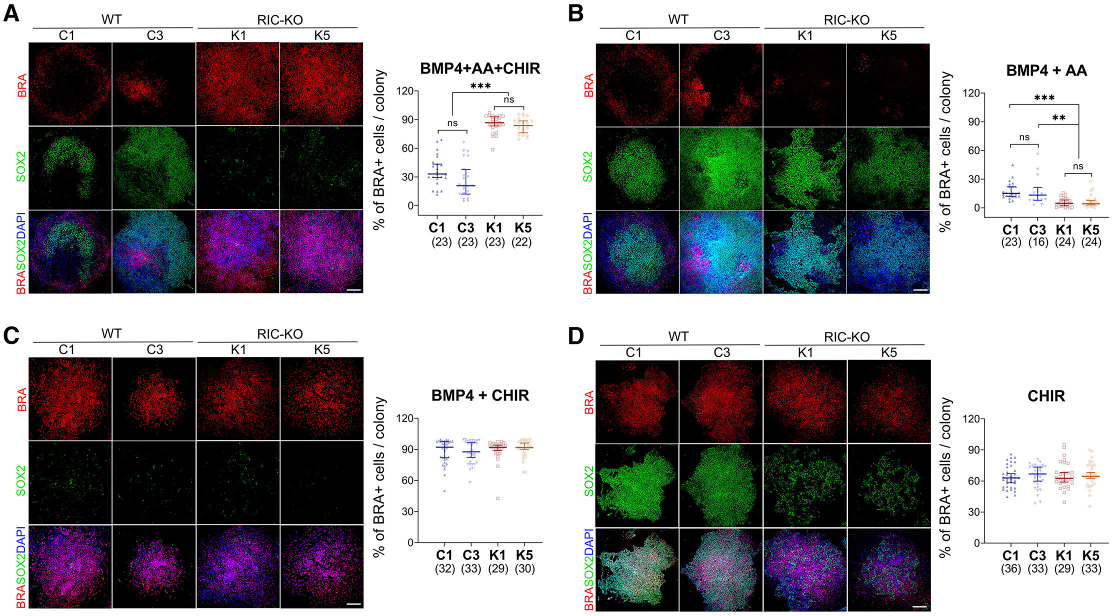

Figure 5. Activation of WNT considerably improves mesendoderm differentiation in RIC-KO hESCs

Immunostaining images of Brachyury (BRA) and SOX2 (left; scale bars, 250 μm) after 48 h of the indicated treatment. Quantification of BRA$^+$ cells (right) is presented as median with 95% confidence interval. Each symbol represents a colony. The total number of colonies analyzed in each line is shown in brackets ($n = 3$). ** and ***$p < 0.005$ and 0.0005, respectively, by one-way ANOVA. (A) BMP4 with Activin A (AA) and CHIR99021 (CHIR); (B) BMP4 and AA; (C) BMP4 and CHIR; (D) CHIR only. See also Figure S5.

suggest that the RIC-KO hESCs are competent to differentiate into mesendoderm and that mTORC2 deficiency possibly disrupts BMP4-induced activation of WNT and/or NODAL pathways. To dissect this further, both hESCs were treated with BMP4 in combination with either CHIR or AA. While AA did not improve BMP4-induced BRA$^+$ cells in RIC-KO cells (Figure 5B), the addition of CHIR dramatically increased BRA$^+$ cells and eliminated the differences between WT and RIC-KO cultures (Figure 5C). Moreover, CHIR alone was sufficient to induce high levels of BRA$^+$ cells in both cultures (Figure 5D). These results imply that mTORC2 deficiency impedes mesendoderm differentiation probably by encumbering BMP4-induced activation of WNT signaling, which is essential for mesendoderm differentiation in hESCs.

Interestingly, when comparing the effects of these treatments within the same cell lines, we observed that AA combined with BMP4 did not significantly increase BRA$^+$ cells compared to BMP4 alone (BMP4 + AA vs. BMP4). However, in WT hESCs, the addition of AA to BMP4 and CHIR (BMP4 + AA + CHIR) reduced the proportion of BRA$^+$ cells compared to BMP4 + CHIR, which was not observed in RIC-KO cells (Figure S5C). The underlying mechanisms behind the context-dependent modulation remain to be elucidated. Together, these results further support the notion that mTORC2 regulates BMP4-induced mesendoderm differentiation possibly through modulating the activation of WNT signaling.

BMP4-induced expression of WNT genes is impeded in RIC-KO hESCs

Given that GSK3 inhibition efficiently induced mesendoderm differentiation in RIC-KO hESCs (Figure 5), we expected that mTORC2 may affect the WNT pathway upstream of GSK3. Indeed, BMP4 significantly upregulated canonical WNT genes, *WNT3*, *WNT3A*, and *WNT8A*, in WT but not in RIC-KO hESCs, and the expression of WNT target genes, *AXIN2* and *LEF1*, was also higher in WT cells (Figures 6A and 6B). Although mTORC2 is known to maximize AKT activity, which may exert inhibitory phosphorylation on GSK3 and thereby activate WNT signaling (Nusse and Clevers 2017), no clear difference was detected on the levels of GSK3 phosphorylation between WT and RIC-KO cells (Figure 6B; Figure S6A). Therefore, mTORC2 deficiency appears to suppress BMP4-induced canonical WNT activation, thereby reducing WNT signaling and impairing mesendoderm differentiation in hESCs.

BMP4 receptors have been reported to be localized at the basolateral sides of cells, meaning that only the peripheral cells of a colony are accessible to externally applied BMP4, and E-CAD has been implicated in modulating WNT signaling patterning in hESCs (Etoc et al., 2016; Martyn

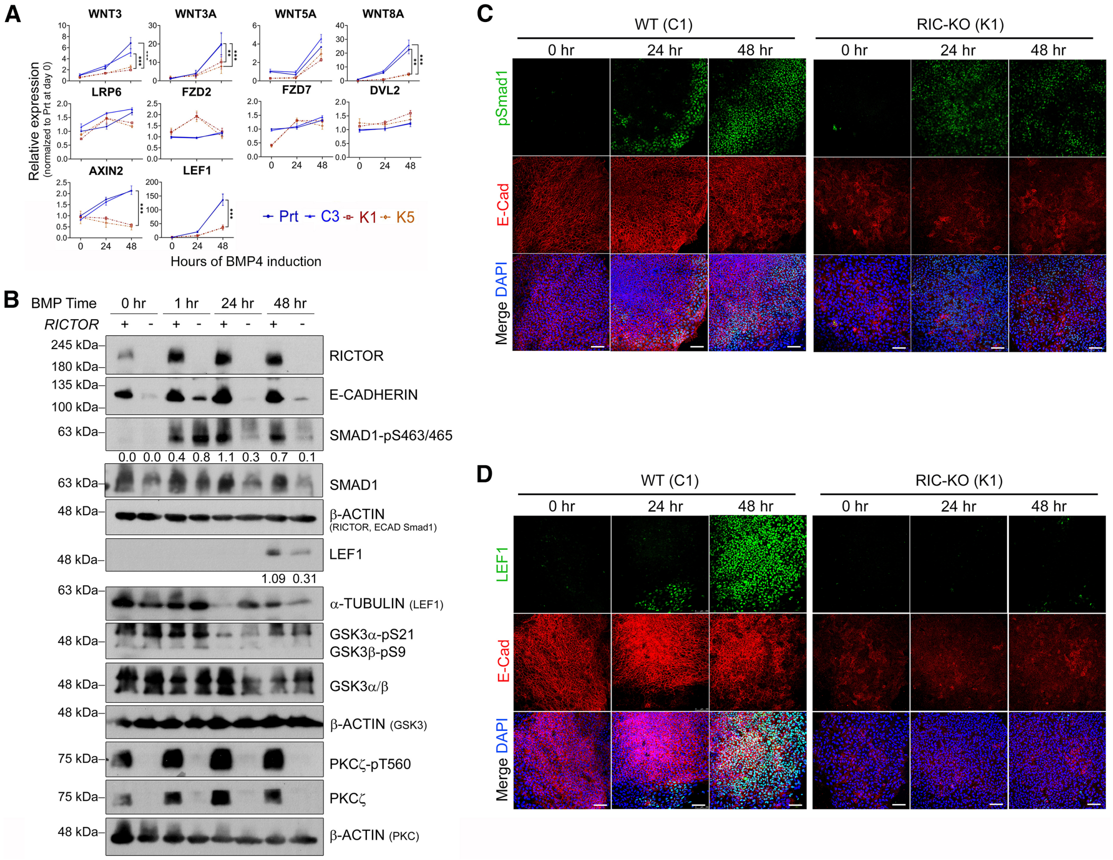

Figure 6. BMP4-induced activation of WNT is hampered in RIC-KO hESCs

(A) Expression of genes associated with WNT signaling during BMP4-induced differentiation by RT-qPCR. Data are presented as mean ± SD ($n = 3$). ** and ***$p < 0.005$ and 0.0005, respectively, by two-way ANOVA.

(B) Immunoblots showing protein expression in WT and RIC-KO hESCs at the indicated time points during BMP4-induced differentiation ($n = 2$). Numbers below the pSmad1 and LEF1 bands indicate the relative signal intensity normalized to the loading control using ImageJ.

(C and D) Immunostaining with the indicated antibodies at the specified time points during BMP4-induced differentiation in C1 and K1 hESCs. Scale bars, 100 μm. ($n = 3$).

See also Figure S6.

et al., 2019; Zhang et al., 2019). Since RIC-KO hESCs exhibited a marked reduction in E-CAD expression and cell interactions (Figure 2), we asked whether these changes could affect BMP4 and WNT signaling. Although both WT and RIC-KO hESCs showed similar acute responses to BMP4, phosphorylation of SMAD1/5/9 (pSMAD1) dramatically declined at 24 h in RIC-KO cells only (Figure 6B). This could be due to higher upregulation of the BMP4 antagonist NOGGIN in RIC-KO hESCs (Figure S6B). Furthermore, the emergence of pSMAD1 signals in WT coincided with the loss of E-CAD within WT colonies (Figure 6C; Figure S6C), and more importantly, LEF1 exhibited a spatial pattern similar to that of pSMAD1 (Figure 6D; Figure S6D). Collectively, these findings suggest that cell-cell interactions may contribute not only to the BMP4 signaling gradient but also to the activation of WNT signaling.

Elimination of cell interactions diminishes BMP4-induced upregulation of WNT genes

To further validate the role of cell-cell interactions in activating WNT genes and mesendoderm differentiation in hESCs, we performed the differentiation using WT hESCs cultured either in colony (CL) or single-cell (SC) form (Figure 7A). As expected, asymmetrically distributed BRA^{+} cells were detected in CL cultures after 48 h of BMP4 treatment, while BRA^{+} cells were almost absent in SC cultures (Figure 7B). In SC cultures, pSMAD1 signals were diffuse, and membrane-bound E-CAD was undetectable (Figure 7C). Expression of canonical WNT ligands, WNT target genes, and mesendoderm markers was lower in SC than in CL cultures (Figure 7D; Figure S7A). However, simultaneous activation of BMP, WNT, and AA pathways in the SC cultures efficiently induced BRA^{+} cells (Figure S7B).

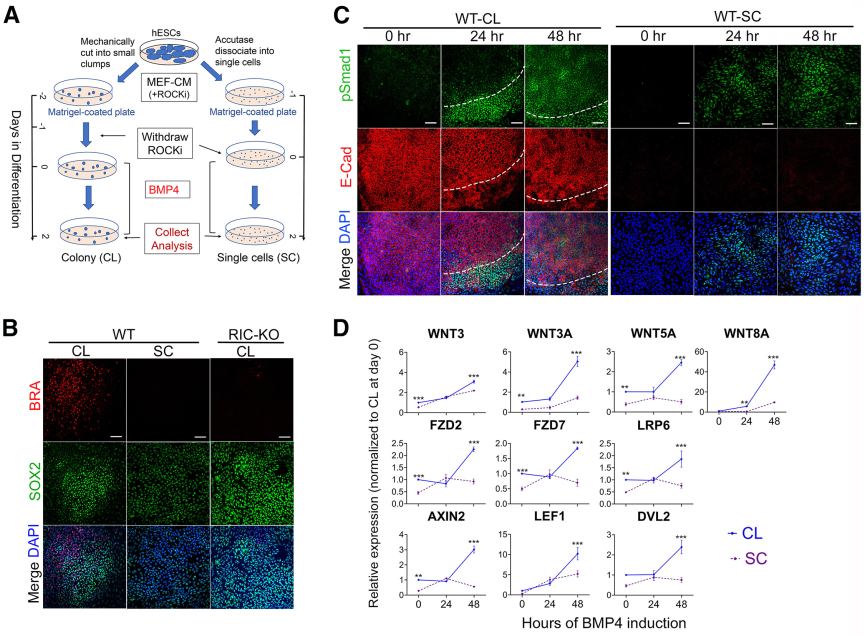

Figure 7. Cell interactions promote BMP4-induced activation of WNT in hESCs
(A) Schematic of the differentiation procedure in WT hESCs as colonies (CLs) and single cells (SCs).
(B) Immunostaining for BRA and SOX2 after 48 h of BMP4 treatment in CL and SC cultures. Scale bars, 100 μm.
(C) Immunostaining for pSMAD1and E-CAD at the indicated time points during BMP4-induced differentiation in CL and SC cultures (n = 3). Scale bars, 100 μm.
(D) Expression of genes associated with WNT signaling during BMP4-induced differentiation in CL and SC cultures by RT-qPCR. Data are presented as mean ± SD (n = 3). ** and ***p < 0.005 and 0.0005, respectively, by two-way ANOVA.
See also Figure S7.

Together, these results support the finding that BMP4-induced mesendoderm differentiation in hESCs requires close cell-cell contact as such interactions are vital for the activation of WNT genes.

DISCUSSION

In this study, we investigated the role of mTORC2 in regulating PSC properties using RIC-DF hESCs. We show that although *RICTOR* disruption initially causes a transient proliferative reduction, the hESCs adapt over time and exhibit enhanced growth without clear genetic alterations. This suggests a capacity for adaptive signaling rewiring, potentially involving upregulation of the fibroblast growth factor (FGF)-MAPK-ERK pathway. Interestingly, this growth recovery is observed only in undifferentiated hESCs while differentiated derivatives (e.g., fibroblast and EB) show growth inhibition, which indicates that the growth recovery reflects adaptive rewiring rather than transformation. Furthermore, our results show that the self-renewal of RIC-KO hESCs can only be maintained in MEF-CM but not in defined mTeSR medium (Chu et al., 2022). It is possible that enriched ECM and extracellular vesicles in MEF-CM compensate, at least partially, for impaired intercellular communication in mTORC2-deficient hESCs (Ahmed and Ffrench-Constant 2016; Kalluri and McAndrews 2023), thereby supporting the maintenance of hESCs. Our results demonstrate that mTORC2 is not essential for the maintenance of hESCs.

Mechanistically, we identify that mTORC2 influences Rho family of GTPases which is crucial for cell adhesion and epithelial morphogenesis (Heasman and Ridley 2008; Jaffe and Hall 2005). In RIC-DF hESCs, increased RhoA-GTP and reduced Rac1-GTP lead to aberrant cell adhesion, which is partially rescued by ROCK inhibition. mTORC2 may regulate these GTPases through multiple pathways, such as protein kinase C (PKC)-mediated phosphorylation of RhoA/Rac regulators, including Rac1-specific guanine nucleotide exchange factor, RhoGDI2, and p190RhoGAP (Griner et al., 2013; Levay et al., 2009; Morrison et al., 2015). As a substrate of mTORC2, PKC is misfolded and degraded upon mTORC2 disruption (Ikenoue et al., 2008), which could affect RhoA/Rac1 signaling. Furthermore, RICTOR has been shown to suppress RhoGDI2 independent of mTORC2 activity to maintain Rac1 GTPase activity (Agarwal et al., 2013).

The main finding of our study is that mTORC2 deficiency in hESCs considerably impairs mesendoderm differentiation, subsequently mesoderm and endoderm formation. Forced activation of the WNT/β-CATENIN pathway fully rescues mesendoderm differentiation in RIC-KO hESCs, underscoring the essential role of WNT signaling in this process (Steinhart and Angers 2018) and indicating that defective WNT signaling might account for the impaired mesendoderm differentiation in RIC-KO hESCs. Importantly, we demonstrate that cell-cell interactions are necessary for BMP-induced expression of canonical WNT ligands in hESCs. While the regulation of WNT signaling has been intensively studied, the research has focused on

downstream components with limited attention to the regulation of upstream WNT ligands. Previous studies have shown that the p53 family directly activates the *Wnt3* gene in mouse ESCs to promote their mesendoderm differentiation (Wang et al., 2017) and that YAP protein can bind to an intragenic enhancer of the *WNT3* gene in hESCs to prevent its premature activation by Activin-induced SMAD2/3 (Estaras et al., 2017). Here, we show that mTORC2-mediated cell interactions are vital for BMP4-induced canonical WNT gene activation and spatial patterning during early differentiation. It may also affect Activin-Smad2/3-induced WNT activation as RIC-KO hESCs show reduced LEF1 to Activin as well. How cell-cell interactions regulate WNT gene activation and whether this is related to p53 or YAP in hESCs await further investigation. Furthermore, reduced epithelial property manifested by low E-CAD in KO hESCs could also affect proper epithelial-to-mesenchymal transition, impacting on mesendoderm/mesoderm differentiation.

Additionally, mTORC2 also differentially modulates BMP and Activin pathways. Specifically, mTORC2 deficiency shortens BMP-Smad1/5 signaling duration but prolongs Activin-Smad2/3 signaling. These effects may be influenced by differences in culture media and the distinct linker sequences between Smad1/5 and Smad2/3 (Macias et al., 2015). Furthermore, mTORC2 deficiency affects MAPK-ERK signaling in a context-dependent manner. RIC-KO hESCs show increased FGF-ERK signaling in MEF-CM but reduced ERK signaling in Activin-induced endoderm cultures, potentially impacting pSmad2/3 signaling (Richardson et al., 2023). Interestingly, while transient mTOR inhibition promotes endoderm formation, complete mTORC2 knockout hinders it, despite sustained Activin-Smad2 signaling. This discrepancy may be explained by the requirement for cell interaction-mediated WNT activation, which is disrupted in RIC-KO cells. Collectively, these findings highlight the integral role of mTORC2 in coordinating multiple signaling pathways during hESC differentiation.

Mesendoderm differentiation recapitulates key aspects of primitive streak formation during gastrulation. Although mTORC2-deficient hESCs exhibit defects in mesendoderm differentiation, mTORC2-null mouse embryos can undergo successful gastrulation and form all three germ layers (Guertin et al., 2006; Shiota et al., 2006). This discrepancy may be attributed to mechanical forces in the rapidly proliferating post-implantation epiblast, which promote cell-cell contact and facilitate signaling required for gastrulation. Additionally, maternal factors present may compensate for mTORC2 loss during early development. Therefore, the role of RICTOR/mTORC2 in differentiation and development is multifaceted and influenced by both embryonic and environmental factors.

METHODS

Culture and maintenance of hESCs

H1 (WA-01) and H7 (WA-07) hESCs from WiCell (https://www.wicell.org/) were routinely cultured on Matrigel-coated plates in hESC culture medium (MEF-CM with 10 ng/mL of bFGF [PeproTech]) with daily medium refreshment. MEF-CM was produced by incubating knockout serum replacement (KSR) medium (20% KSR, 1 mM L-glutamine, 1% non-essential amino acid, 0.1 M β-mercaptoethanol, 1% Penicillin-Streptomycin, and 4 ng/mL bFGF in knockout DMEM) on MEF for 24 h (Noisa et al., 2011). hESCs were split at a 1:3 ratio by collagenase-assisted mechanical method. When required, hESCs were also dissociated into single cells by Accutase (Merck). All reagents were from Thermo Fisher Scientific unless stated. All hESC lines are negative for mycoplasma by regular test.

Generation of RIC-KO and RIC-KD hESCs

Targeting vector eSpCas9(1.1)-2A-Puro was constructed by replacing the Cas9 in pSpCas9(BB)-2A-Puro (PX459) with Cas9(1.1) from eSpCas9(1.1) (Addgene) (Ran et al., 2013; Slaymaker et al., 2016). Two sgRNAs (GGCCACAGTGAAG AAAAACTGGG and AGACTCCAGTATTCTCCAGA-AGG) were selected for co-transfection into H1 hESCs using the Neon Transfection system (Thermo Fisher Scientific) following the manufacturer's instruction (Figures S1A and S1B). Successful targeted colonies were selected through two rounds of colony picking and screening. WT control clones were established in the same way but without sgRNA.

RIC-KD hESCs were established by transducing H7 hESCs with lentivirus expressing Rictor_2 shRNA (Addgene) as previously described (Sarbassov et al., 2005; Yu et al., 2015). Transduced cells were selected with puromycin (5 μg/mL) for a week from 48 h post infection.

Differentiation via EB formation

EB formation was performed using AggreWell 400 plate (STEMCELL Technologies). The plate was prepared with anti-adherent rinsing solution and centrifuged at 1,300 g for 5 min. After removal of the solution, 7×10^5 to 8×10^5 hESCs/well were seeded in EB medium (DMEM/F12 containing 20% fetal bovine serum or KSR, 1 mM glutamine, 1% Penicillin-Streptomycin, 1:100 N2, and 1:200 B27) with 10 μM ROCKi and centrifuged at 100 g for 3 min. Cell aggregates were gently transferred to low-attachment plates next day and cultured in suspension for another 6 days with medium change every 2 days. EBs were then plated to adherent culture dishes for further 7 days (Figure S2E).

BMP4-induced mesendoderm differentiation

In colony, the method was adapted from previous ones (Bernardo et al., 2011; Warmflash et al., 2014). hESCs were dissociated into small clusters and seeded at a 1:5 ratio in hESC culture medium with 10 μM ROCKi that was withdrawn the next day. Differentiation started by adding 50 ng/mL BMP4 with or without 50 ng/mL AA and/or 6 μM CHIR into hESC culture medium for 48 h (Figure S4E).

In spheroids, 5×10^5 hESCs/well were seeded into AggreWell400 plate in hESC culture medium with 10 μM ROCKi and centrifuged at 100 g for 3 min. The medium was refreshed next day with 1 ng/mL BMP4 and 10 μM ROCKi and cultured for 48 h (Figure S4D).

In single cells, hESCs were seeded on Matrigel-coated plates at $1 \times 10^4/cm^2$ in hESC culture medium with 10 μM ROCKi. Differentiation started the next day with fresh medium containing 50 ng/mL BMP4 for 48 h (Figure 7A).

AA-induced endoderm differentiation

hESCs were seeded on Matrigel-coated plates at $5 \times 10^4/cm^2$ in hESC culture medium with 10 μM ROCKi. Differentiation started by replacing hESC culture medium with RPMI1640/B27 medium containing 100 ng/mL AA for 72 h with daily medium refreshing (Yu et al., 2015) (Figure S4C).

Cell adhesion assay

hESCs were seeded at 2.5×10^4 cells/well on a Matrigel-coated 96-well plate in hESC culture medium containing 2.5–10 μM ROCKi and incubated at 37°C, 5% CO_2 for 10 min. The attached cells were stained with 200 μL 0.2% crystal violet solution (Merck) for 10 min. After washing, 1% SDS (100 μL/well) was added and incubated for 15 min on a shaker, and the data were recorded at 570 nm wavelength on a microplate reader (Bio-Rad).

Alkaline phosphatase colony formation assay and CCK8 assay

hESCs were seeded at 6×10^3 hESCs/well in 12-well plates in hESC culture medium for 5 days before stained with Leukocyte Alkaline Phosphatase Kit (Merck) following manufacturer's instruction. Cell proliferation was measured using Cell Counting Kit-8 (CCK-8) from APExBIO following the manufacturer's instructions and using a microplate reader.

Reverse-transcription qPCR

Total RNAs were isolated with TRI-Reagent (Merck), and cDNA was synthesized with Superscript II reverse transcriptase (NEB). qPCR was performed in a StepOnePlus System (Applied BioSystems) using SYBR Green Jumpstart Taq Ready Mix (Merck). Table S4 lists all the primers.

RNA-seq analysis

Total RNA was extracted using TRIzolTM Plus RNA Purification Kit (Thermo Fisher Scientific). Library preparation, sequencing, and initial quality check were performed by Imperial BRC Genomics Facility using Illumina HiSeq 4000. Raw fastq data were trimmed by Fastp v.0.23.2 (Chen et al., 2018), and STAR v.2.7.10a (Dobin et al., 2013) was used to create genome mapping indices with human genome files (GRCh38 release 104). Transcripts were quantified by RSEM v.1.3.3 (Li and Dewey 2011). All downstream analyses were carried out in R v.4.1.2 using Bioconductor packages with variance differences and batch effects were corrected (Ritchie et al., 2015). Expression analysis was performed by DESeq2 v.1.34.0 (Love et al., 2014) with a false discovery rate threshold of 0.05. Genes with a p-adj value < 0.05 and a log2 fold change > 1 were considered differentially expressed. GO and KEGG pathway analyses were performed separately on upregulated and downregulated genes using clusterProfiler v.4.2.2 in R (Yu et al., 2012). p values were adjusted using the Benjamini-Hochberg method with a cutoff at 0.05.

Immunoblotting

Cell lysates were obtained in RIPA (Radio Immunoprecipitation Assay) buffer supplemented with phosphatase and protease inhibitors (all from Merck). 10–25 μg proteins were resolved in 8% SDS-PAGE gels and blotted onto polyvinylidene membranes, which were probed with primary antibodies overnight at 4°C followed by horseradish peroxidase-conjugated secondary antibodies for 1 h before signals detected by ECL (enhanced chemi-luminescent) and exposed onto CL-XPosure film (Thermo Fisher Scientific). Table S5 lists all the antibodies.

Immunostaining and image analysis

hESCs cultured on Nunc Thermanox Coverslips (Thermo Fisher Scientific) or differentiated spheroids were fixed for 20 min with 4% paraformaldehyde. After incubation with blocking buffer, cells were probed with primary antibodies overnight at 4°C, followed by 1 h incubation with fluorescent conjugated secondary antibodies before counterstained with 4,6-diamidino-2-phenylindol (Merck) and mounted onto microscope slides with Mowiol 4-88 solution. All the images, except those in Figure S4A, were captured with a Leica SP5 II confocal microscope or Leica DM5500 fluorescent microscope. The images in Figure S4A were taken with Nikon Eclipse E400 microscope. Quantification of positive signals was performed using ImageJ and CellProfiler (McQuin et al., 2018) software after subtracting background signals using the rolling ball method with a radius of 50 pixels.

RhoA activation assay and Rac1-GTP assay

RhoA activation assay was performed using RhoA G-LISA Activation Assay Kit – colorimetric format (Cytoskeleton, Inc.) following manufacturer's instruction. The signals were recorded as absorbance at 490 nm wavelength using microplate spectrophotometer. Rac1-GTP assay was performed using Non-radioactive Rac1 Activation Assay Kit (Merck) following manufacturer's instruction.

Statistical analysis

All data are presented as the means with error bars indicating standard deviation (SD), unless indicated otherwise, from at least three biological replicates. The "*n*" in the figure legends and text represent number of independent experiments, e.g., differentiation or treatments. The *p* values were obtained by unpaired two-tail *t* test and one-way or two-way ANOVA using GraphPad Prism 9 except RNA-seq analysis.

RESOURCE AVAILABILITY

Lead contact

Further information and requests for resources and reagents should be directed to and will be fulfilled by the lead contact, Wei Cui (wei.cui@imperial.ac.uk).

Materials availability

The RIC-RO hESC lines and other materials generated and used in this study will be available from the lead contact upon request.

Data and code availability

The RNA-seq datasets of WT and RIC-KO hESCs have been deposited in the Gene Expression Omnibus database accession (GSE243723) and are publicly available.

ACKNOWLEDGMENTS

This research was supported by Genesis Research Trust, United Kingdom (grant nos. P64247 and PA9387). F.B. is supported by PhD scholarship from the Punjab Educational Endowment Fund, and Y.-H.C. is partially sponsored by GSSA scholarship. We thank the Imperial BRC Genomics Facility (IGF) for providing the RNA sequencing service and all the members in Stem Cell Differentiation group for their helpful discussion.

AUTHOR CONTRIBUTIONS

W.C. conceived the project and designed research strategy and experiments. Y.-H.C. and F.B. designed gene targeting strategies and generated RIC-KO and -KD hESC lines, respectively, and carried out initial characterization on them. L.T., F.B., and P.D.W. performed differentiation experiments and data analysis. L.T., F.B., and X.M. performed cell culture, molecular experiments, and data analysis. L.T. performed RNA-seq and analysis, supervised by S.A. Y.Z. provided technical support for cell culture and molecular experiments. W.C. and L.T. wrote the manuscript with inputs from all authors.

DECLARATION OF INTERESTS

The authors declare no competing interests.

SUPPLEMENTAL INFORMATION

Supplemental information can be found online at https://doi.org/10.1016/j.stemcr.2025.102680.

Received: September 13, 2024
Revised: September 19, 2025
Accepted: September 19, 2025
Published: October 16, 2025

REFERENCES

Agarwal, N.K., Chen, C.H., Cho, H., Boulbès, D.R., Spooner, E., and Sarbassov, D.D. (2013). Rictor regulates cell migration by suppressing RhoGDI2. Oncogene *32*, 2521–2526. https://doi.org/10.1038/onc.2012.287.

Ahmed, M., and Ffrench-Constant, C. (2016). Extracellular Matrix Regulation of Stem Cell Behavior. Curr. Stem Cell Rep. *2*, 197–206. https://doi.org/10.1007/s40778-016-0056-2.

Aimi, F., Georgiopoulou, S., Kalus, I., Lehner, F., Hegglin, A., Limani, P., Gomes de Lima, V., Rüegg, M.A., Hall, M.N., Lindenblatt, N., et al. (2015). Endothelial Rictor is crucial for midgestational development and sustained and extensive FGF2-induced neovascularization in the adult. Sci. Rep. *5*, 17705. https://doi.org/10.1038/srep17705.

Aylett, C.H.S., Sauer, E., Imseng, S., Boehringer, D., Hall, M.N., Ban, N., and Maier, T. (2016). Architecture of human mTOR complex 1. Science *351*, 48–52. https://doi.org/10.1126/science.aaa3870.

Battaglioni, S., Benjamin, D., Wälchli, M., Maier, T., and Hall, M.N. (2022). mTOR substrate phosphorylation in growth control. Cell *185*, 1814–1836. https://doi.org/10.1016/j.cell.2022.04.013.

Bentzinger, C.F., Romanino, K., Cloëtta, D., Lin, S., Mascarenhas, J. B., Oliveri, F., Xia, J., Casanova, E., Costa, C.F., Brink, M., et al. (2008). Skeletal muscle-specific ablation of raptor, but not of rictor, causes metabolic changes and results in muscle dystrophy. Cell Metab. *8*, 411–424. https://doi.org/10.1016/j.cmet.2008.10.002.

Bernardo, A.S., Faial, T., Gardner, L., Niakan, K.K., Ortmann, D., Senner, C.E., Callery, E.M., Trotter, M.W., Hemberger, M., Smith, J.C., et al. (2011). BRACHYURY and CDX2 mediate BMP-induced differentiation of human and mouse pluripotent stem cells into embryonic and extraembryonic lineages. Cell Stem Cell *9*, 144–155. https://doi.org/10.1016/j.stem.2011.06.015.

Bulut-Karslioglu, A., Biechele, S., Jin, H., Macrae, T.A., Hejna, M., Gertsenstein, M., Song, J.S., and Ramalho-Santos, M. (2016). Inhibition of mTOR induces a paused pluripotent state. Nature *540*, 119–123. https://doi.org/10.1038/nature20578.

Burridge, K., and Wennerberg, K. (2004). Rho and Rac take center stage. Cell *116*, 167–179. https://doi.org/10.1016/s0092-8674(04)00003-0.

Chen, S., Zhou, Y., Chen, Y., and Gu, J. (2018). "fastp: an ultra-fast all-in-one FASTQ preprocessor". Bioinformatics *34*, i884–i890. https://doi.org/10.1093/bioinformatics/bty560.

Chu, Q., Liu, F., He, Y., Jiang, X., Cai, Y., Wu, Z., Yan, K., Geng, L., Zhang, Y., Feng, H., et al. (2022). mTORC2/RICTOR exerts differential levels of metabolic control in human embryonic, mesenchymal and neural stem cells. Protein Cell *13*, 676–682. https://doi.org/10.1007/s13238-021-00898-9.

Cui, G., Feng, S., Yan, Y., Wang, L., He, X., Li, X., Duan, Y., Chen, J., Tang, K., Zheng, P., et al. (2022). Spatial molecular anatomy of germ layers in the gastrulating cynomolgus monkey embryo. Cell Rep. *40*, 111285. https://doi.org/10.1016/j.celrep.2022.111285.

Cybulski, N., Polak, P., Auwerx, J., Rüegg, M.A., and Hall, M.N. (2009). mTOR complex 2 in adipose tissue negatively controls whole-body growth. Proc. Natl. Acad. Sci. USA *106*, 9902–9907. https://doi.org/10.1073/pnas.0811321106.

Dobin, A., Davis, C.A., Schlesinger, F., Drenkow, J., Zaleski, C., Jha, S., Batut, P., Chaisson, M., and Gingeras, T.R. (2013). STAR: ultrafast universal RNA-seq aligner. Bioinformatics *29*, 15–21. https://doi.org/10.1093/bioinformatics/bts635.

Dragert, K., Bhattacharya, I., Pellegrini, G., Seebeck, P., Azzi, A., Brown, S.A., Georgiopoulou, S., Held, U., Blyszczuk, P., Arraset al, M., and Haas, E. (2015). Deletion of Rictor in brain and fat alters peripheral clock gene expression and increases blood pressure. Hypertension *66*, 332–339. https://doi.org/10.1161/HYPERTENSIONAHA.115.05398.

Estaras, C., Hsu, H.T., Huang, L., and Jones, K.A. (2017). YAP repression of the WNT3 gene controls hESC differentiation along the cardiac mesoderm lineage. Genes & development *31*, 2250–2263. https://doi.org/10.1101/gad.307512.117.

Etoc, F., Metzger, J., Ruzo, A., Kirst, C., Yoney, A., Ozair, M.Z., Brivanlou, A.H., and Siggia, E.D. (2016). A Balance between Secreted Inhibitors and Edge Sensing Controls Gastruloid Self-Organization. Dev. Cell *39*, 302–315. https://doi.org/10.1016/j.devcel.2016.09.016.

Godel, M., Hartleben, B., Herbach, N., Liu, S., Zschiedrich, S., Lu, S., Debreczeni-Mor, A., Lindenmeyer, M.T., Rastaldi, M.P., Hartlebenet al, G., and Huber, T.B. (2011). Role of mTOR in podocyte function and diabetic nephropathy in humans and mice. J. Clin. Investig. *121*, 2197–2209. https://doi.org/10.1172/JCI44774.

Griner, E.M., Churchill, M.E.A., Brautigan, D.L., and Theodorescu, D. (2013). PKCalpha phosphorylation of RhoGDI2 at Ser31 disrupts interactions with Rac1 and decreases GDI activity. Oncogene *32*, 1010–1017. https://doi.org/10.1038/onc.2012.124.

Guertin, D.A., Stevens, D.M., Thoreen, C.C., Burds, A.A., Kalaany, N.Y., Moffat, J., Brown, M., Fitzgerald, K.J., and Sabatini, D.M. (2006). Ablation in mice of the mTORC components raptor, rictor, or mLST8 reveals that mTORC2 is required for signaling to Akt-FOXO and PKCalpha, but not S6K1. Dev. Cell *11*, 859–871. https://doi.org/10.1016/j.devcel.2006.10.007.

Hagiwara, A., Cornu, M., Cybulski, N., Polak, P., Betz, C., Trapani, F., Terracciano, L., Heim, M.H., Rüegg, M.A., and Hall, M.N. (2012). Hepatic mTORC2 activates glycolysis and lipogenesis through Akt, glucokinase, and SREBP1c. Cell Metab. *15*, 725–738. https://doi.org/10.1016/j.cmet.2012.03.015.

Heasman, S.J., and Ridley, A.J. (2008). Mammalian Rho GTPases: new insights into their functions from *in vivo* studies. Nat. Rev. Mol. Cell Biol. *9*, 690–701. https://doi.org/10.1038/nrm2476.

Ikenoue, T., Inoki, K., Yang, Q., Zhou, X., and Guan, K.L. (2008). Essential function of TORC2 in PKC and Akt turn motif phosphorylation, maturation and signalling. EMBO J. *27*, 1919–1931. https://doi.org/10.1038/emboj.2008.119.

Jaffe, A.B., and Hall, A. (2005). Rho GTPases: biochemistry and biology. Annu. Rev. Cell Dev. Biol. *21*, 247–269. https://doi.org/10.1146/annurev.cellbio.21.020604.150721.

Kalluri, R., and McAndrews, K.M. (2023). The role of extracellular vesicles in cancer. Cell *186*, 1610–1626. https://doi.org/10.1016/j.cell.2023.03.010.

Kim, J., and Guan, K.L. (2019). mTOR as a central hub of nutrient signalling and cell growth. Nat. Cell Biol. *21*, 63–71. https://doi.org/10.1038/s41556-018-0205-1.

Lamming, D.W., Ye, L., Katajisto, P., Goncalves, M.D., Saitoh, M., Stevens, D.M., Davis, J.G., Salmon, A.B., Richardson, A., Ahima, R.S., et al. (2012). Rapamycin-induced insulin resistance is mediated by mTORC2 loss and uncoupled from longevity. Science *335*, 1638–1643. https://doi.org/10.1126/science.1215135.

Levay, M., Settleman, J., and Ligeti, E. (2009). Regulation of the substrate preference of p190RhoGAP by protein kinase C-mediated phosphorylation of a phospholipid binding site. Biochemistry *48*, 8615–8623. https://doi.org/10.1021/bi900667y.

Li, B., and Dewey, C.N. (2011). RSEM: accurate transcript quantification from RNA-Seq data with or without a reference genome. BMC Bioinf. *12*, 323. https://doi.org/10.1186/1471-2105-12-323.

Liu, G.Y., and Sabatini, D.M. (2020). mTOR at the nexus of nutrition, growth, ageing and disease. Nat. Rev. Mol. Cell Biol. *21*, 183–203. https://doi.org/10.1038/s41580-019-0199-y.

Love, M.I., Huber, W., and Anders, S. (2014). Moderated estimation of fold change and dispersion for RNA-seq data with DESeq2. Genome Biol. *15*, 550. https://doi.org/10.1186/s13059-014-0550-8.

Macias, M.J., Martin-Malpartida, P., and Massagué, J. (2015). Structural determinants of Smad function in TGF-beta signaling. Trends Biochem. Sci. *40*, 296–308. https://doi.org/10.1016/j.tibs.2015.03.012.

Martin, E., Ouellette, M.H., and Jenna, S. (2016). Rac1/RhoA antagonism defines cell-to-cell heterogeneity during epidermal morphogenesis in nematodes. J. Cell Biol. *215*, 483–498. https://doi.org/10.1083/jcb.201604015.

Martyn, I., Brivanlou, A.H., and Siggia, E.D. (2019). A wave of WNT signaling balanced by secreted inhibitors controls primitive streak formation in micropattern colonies of human embryonic stem cells. Development *146*, dev172791. https://doi.org/10.1242/dev.172791.

McQuin, C., Goodman, A., Chernyshev, V., Kamentsky, L., Cimini, B.A., Karhohs, K.W., Doan, M., Ding, L., Rafelski, S.M., Thirstrup, D., et al. (2018). CellProfiler 3.0: Next-generation image processing for biology. PLoS Biol. *16*, e2005970. https://doi.org/10.1371/journal.pbio.2005970.

Morrison, M.M., Young, C.D., Wang, S., Sobolik, T., Sanchez, V.M., Hicks, D.J., Cook, R.S., and Brantley-Sieders, D.M. (2015). mTOR Directs Breast Morphogenesis through the PKC-alpha-Rac1 Signaling Axis. PLoS Genet. *11*, e1005291. https://doi.org/10.1371/journal.pgen.1005291.

Mukherjee, S., Chaturvedi, P., Rankin, S.A., Fish, M.B., Wlizla, M., Paraiso, K.D., MacDonald, M., Chen, X., Weirauch, M.T., Blitz, I.L., et al. (2020). Sox17 and beta-catenin co-occupy Wnt-responsive enhancers to govern the endoderm gene regulatory network. eLife *9*, e58029. https://doi.org/10.7554/eLife.58029.

Noisa, P., Urrutikoetxea-Uriguen, A., and Cui, W. (2011). Generation of human embryonic stem cells carrying lineage specific reporters. Methods Mol. Biol. *690*, 95–106. https://doi.org/10.1007/978-1-60761-962-8_6.

Nowotschin, S., and Hadjantonakis, A.K. (2020). Guts and gastrulation: Emergence and convergence of endoderm in the mouse embryo. Curr. Top. Dev. Biol. *136*, 429–454. https://doi.org/10.1016/bs.ctdb.2019.11.012.

Nusse, R., and Clevers, H. (2017). Wnt/beta-Catenin Signaling, Disease, and Emerging Therapeutic Modalities. Cell *169*, 985–999. https://doi.org/10.1016/j.cell.2017.05.016.

Ran, F.A., Hsu, P.D., Wright, J., Agarwala, V., Scott, D.A., and Zhang, F. (2013). Genome engineering using the CRISPR-Cas9 system. Nat. Protoc. *8*, 2281–2308. https://doi.org/10.1038/nprot.2013.143.

Richardson, L., Wilcockson, S.G., Guglielmi, L., and Hill, C.S. (2023). Context-dependent TGFbeta family signalling in cell fate regulation. Nat. Rev. Mol. Cell Biol. *24*, 876–894. https://doi.org/10.1038/s41580-023-00638-3.

Ritchie, M.E., Phipson, B., Wu, D., Hu, Y., Law, C.W., Shi, W., and Smyth, G.K. (2015). "limma powers differential expression analyses for RNA-sequencing and microarray studies. Nucleic Acids Res. *43*, e47. https://doi.org/10.1093/nar/gkv007.

Rodaway, A., and Patient, R. (2001). Mesendoderm. an ancient germ layer? Cell *105*, 169–172. https://doi.org/10.1016/s0092-8674(01)00307-5.

Rojas, A., De Val, S., Heidt, A.B., Xu, S.M., Bristow, J., and Black, B.L. (2005). Gata4 expression in lateral mesoderm is downstream of BMP4 and is activated directly by Forkhead and GATA transcription factors through a distal enhancer element. Development *132*, 3405–3417. https://doi.org/10.1242/dev.01913.

Sarbassov, D.D., Guertin, D.A., Ali, S.M., and Sabatini, D.M. (2005). Phosphorylation and regulation of Akt/PKB by the rictor-mTOR complex. Science *307*, 1098–1101. https://doi.org/10.1126/science.1106148.

Scaiola, A., Mangia, F., Imseng, S., Boehringer, D., Berneiser, K., Shimobayashi, M., Stuttfeld, E., Hall, M.N., Ban, N., and Maier, T. (2020). The 3.2-A resolution structure of human mTORC2. Sci. Adv. *6*, eabc1251. https://doi.org/10.1126/sciadv.abc1251.

Shiota, C., Woo, J.T., Lindner, J., Shelton, K.D., and Magnuson, M.A. (2006). Multiallelic disruption of the rictor gene in mice reveals that mTOR complex 2 is essential for fetal growth and viability. Dev. Cell *11*, 583–589. https://doi.org/10.1016/j.devcel.2006.08.013.

Simunovic, M., Metzger, J.J., Etoc, F., Yoney, A., Ruzo, A., Martyn, I., Croft, G., You, D.S., Brivanlou, A.H., and Siggia, E.D. (2019). A 3D model of a human epiblast reveals BMP4-driven symmetry breaking. Nat. Cell Biol. *21*, 900–910. https://doi.org/10.1038/s41556-019-0349-7.

Slaymaker, I.M., Gao, L., Zetsche, B., Scott, D.A., Yan, W.X., and Zhang, F. (2016). Rationally engineered Cas9 nucleases with improved specificity. Science *351*, 84–88. https://doi.org/10.1126/science.aad5227.

Steinhart, Z., and Angers, S. (2018). Wnt signaling in development and tissue homeostasis. Development *145*, dev146589. https://doi.org/10.1242/dev.146589.

Wang, Q., Zou, Y., Nowotschin, S., Kim, S.Y., Li, Q.V., Soh, C.L., Su, J., Zhang, C., Shu, W., Xi, Q., et al. (2017). The p53 Family Coordinates Wnt and Nodal Inputs in Mesendodermal Differentiation of Embryonic Stem Cells. Cell Stem Cell *20*, 70–86. https://doi.org/10.1016/j.stem.2016.10.002.

Warmflash, A., Sorre, B., Etoc, F., Siggia, E.D., and Brivanlou, A.H. (2014). A method to recapitulate early embryonic spatial patterning in human embryonic stem cells. Nat. Methods *11*, 847–854. https://doi.org/10.1038/nmeth.3016.

Weidinger, G., Thorpe, C.J., Wuennenberg-Stapleton, K., Ngai, J., and Moon, R.T. (2005). The Sp1-related transcription factors sp5 and sp5-like act downstream of Wnt/beta-catenin signaling in mesoderm and neuroectoderm patterning. Curr. Biol. *15*, 489–500. https://doi.org/10.1016/j.cub.2005.01.041.

Yang, Q., Inoki, K., Ikenoue, T., and Guan, K.L. (2006). Identification of Sin1 as an essential TORC2 component required for complex formation and kinase activity. Genes Dev. *20*, 2820–2832. https://doi.org/10.1101/gad.1461206.

Yu, G., Wang, L.G., Han, Y., and He, Q.Y. (2012). clusterProfiler: an R package for comparing biological themes among gene clusters. OMICS *16*, 284–287. https://doi.org/10.1089/omi.2011.0118.

Yu, J.S.L., Ramasamy, T.S., Murphy, N., Holt, M.K., Czapiewski, R., Wei, S.K., and Cui, W. (2015). PI3K/mTORC2 regulates TGF-beta/Activin signalling by modulating Smad2/3 activity via linker phosphorylation. Nat. Commun. *6*, 7212. https://doi.org/10.1038/ncomms8212.

Zhang, Z., Zwick, S., Loew, E., Grimley, J.S., and Ramanathan, S. (2019). Mouse embryo geometry drives formation of robust signaling gradients through receptor localization. Nat. Commun. *10*, 4516. https://doi.org/10.1038/s41467-019-12533-7.

Zhao, X., Lu, S., Nie, J., Hu, X., Luo, W., Wu, X., Liu, H., Feng, Q., Chang, Z., Liu, Y., et al. (2014). Phosphoinositide-dependent kinase 1 and mTORC2 synergistically maintain postnatal heart growth and heart function in mice. Mol. Cell Biol. *34*, 1966–1975. https://doi.org/10.1128/MCB.00144-14.

Zhou, J., Su, P., Wang, L., Chen, J., Zimmermann, M., Genbacev, O., Afonja, O., Horne, M.C., Tanaka, T., Duan, E., et al. (2009). mTOR supports long-term self-renewal and suppresses mesoderm and endoderm activities of human embryonic stem cells. Proc. Natl. Acad. Sci. USA *106*, 7840–7845. https://doi.org/10.1073/pnas.0901854106.

Stem Cell Reports, Volume *20*

Supplemental Information

mTORC2-mediated cell-cell interactions promote BMP4-induced WNT activation and mesoderm differentiation

Li Tong, Faiza Batool, Yueh-Ho Chiu, Priscilla Di Wu, Yudong Zhou, Xiaolun Ma, Santosh Atanur, and Wei Cui

SUPPLEMENTAL INFORMATION

Figure S1

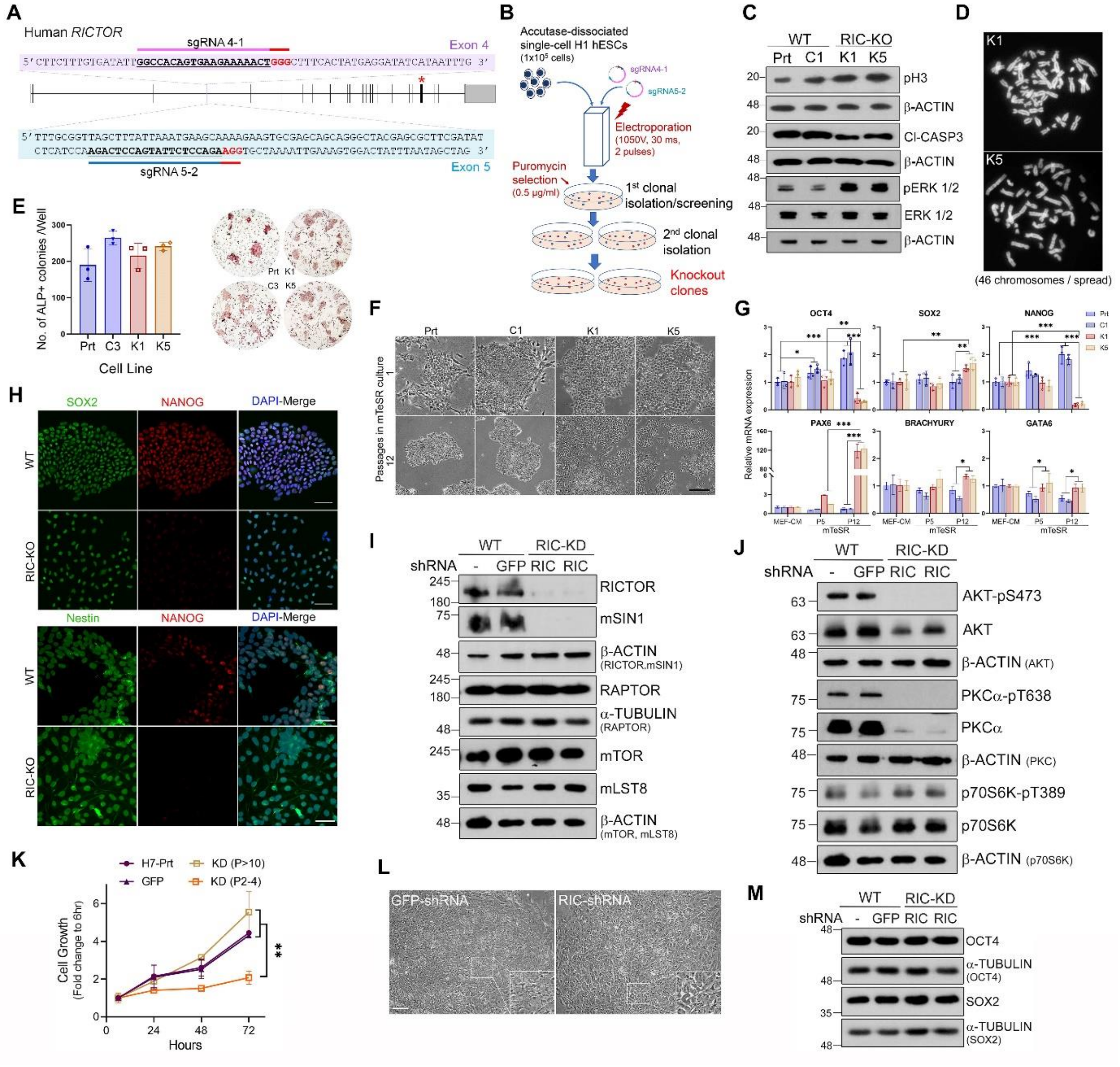

Figure S1. Generation of RICTOR-knockout and -knockdown hESCs.

(A) Diagram depicting human *RICTOR* gene, sgRNA targeting sequences and shRNA site (* in red).

(B) Schematic of generating *RICTOR*-KO clonal hESC lines.

(C) Representative immunoblots of the proliferation marker pH3 and apoptotic marker cleaved caspase 3 (Cl-CASP3) as well as ERK1/2-pThr202/Tyr204 in WT and RIC-KO hESCs (n = 3).

(D) Representative images of metaphase spreads of RIC-KO clones (n = 7 per line).

(E) Alkaline phosphatase colony formation assay. Quantitative analysis (left) shown as mean ± SD (n = 3) and representative images (right). Prt, C3 and K1, K5 represent H1 parental, control clone 3 and RIC-KO clone 1 and 5, respectively.

(F) Representative images of WT and RIC-KO hESCs in mTeSR cultures at passage 1 and 12. Scale bar = 200 μm).

(G) Comparison of mRNA expression in WT and RIC-KO hESCs under indicated culture conditions. Data are presented as mean ± SD (n = 3).

(H) Immunostaining with indicated antibodies on WT and RIC-KO hESCs cultured under mTeSR conditions for 12 passages. Scale bar = 50 μm.

(I) Immunoblots of RICTOR and mTOR complexes protein subunits in RICTOR-knockdown (RIC-KD) H7 hESCs.

(J) Immunoblots show abolished mTORC2 activities in RIC-KD H7 hESCs.

(K) Proliferation of WT and RIC-KD H7 hESCs by CCK8 assay. Indicated passage numbers of H7 RIC-KD are counted after selection. Data presented as mean ± SEM (n = 3).

(L) Representative phase-contrast images of H7 WT and RIC-KD hESCs. Scale bar = 100 μm.

(M) Immunoblots of key pluripotent transcription factors in WT and RIC-KD hESCs. All the immunoblotting experiments are repeated at least once with different culture passages (n ≥ 2).

*, ** & ***, p <0.05, 0.005 & 0.0005, respectively by two-way ANOVA.

Related to Figure 1.

Figure S2

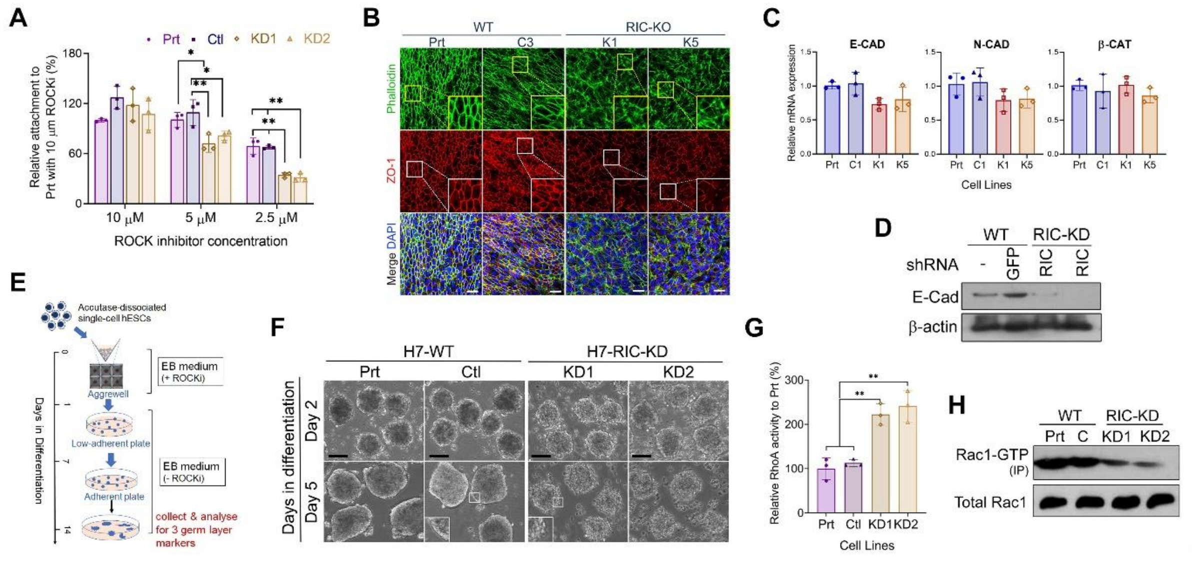

Figure S2. Effects on cell adhesion in RIC-KO and RIC-KD hESCs.

(A) Cell attachment assay in RIC-KD H7 hESCs as described in Figure 2B.

(B) Expression of ZO1 and F-ACTIN (Phalloidin) in H1 RIC-KO hESCs by immunostaining. Scale bar = 50 µm. For the enlarged inserts, the scale bar = 25 µm.

(C) RT-qPCR showing expression of adhesion complex genes, β-CATENIN (β-CAT), E- and N-CADHERIN (E-CAD and N-CAD) in H1 RIC-KO hESCs. Data are presented as mean ± SD (n = 3).

(D) E-CADHERIN expression in H7 RIC-KD and WT hESCs by immunoblotting (n = 2).

(E) Schematic illustrating the procedure of EB formation.

(F) Representative images of H7 RIC-KD EB formation in the presence of ROCKi. Insert showing blebs in RIC-KD EBs. Scale bar = 100 µm.

(G) RhoA activity in H7 WT and RIC-KD hESCs. Data are presented as mean ± SD (n = 3). ** $p < 0.005$ by one-way ANOVA.

(H) Rac1-GTP in WT and RIC-KD H7 hESCs (n = 2). KD1 & KD2 are derived from two knockdown experiments.

Related to Figure 2

Figure S3

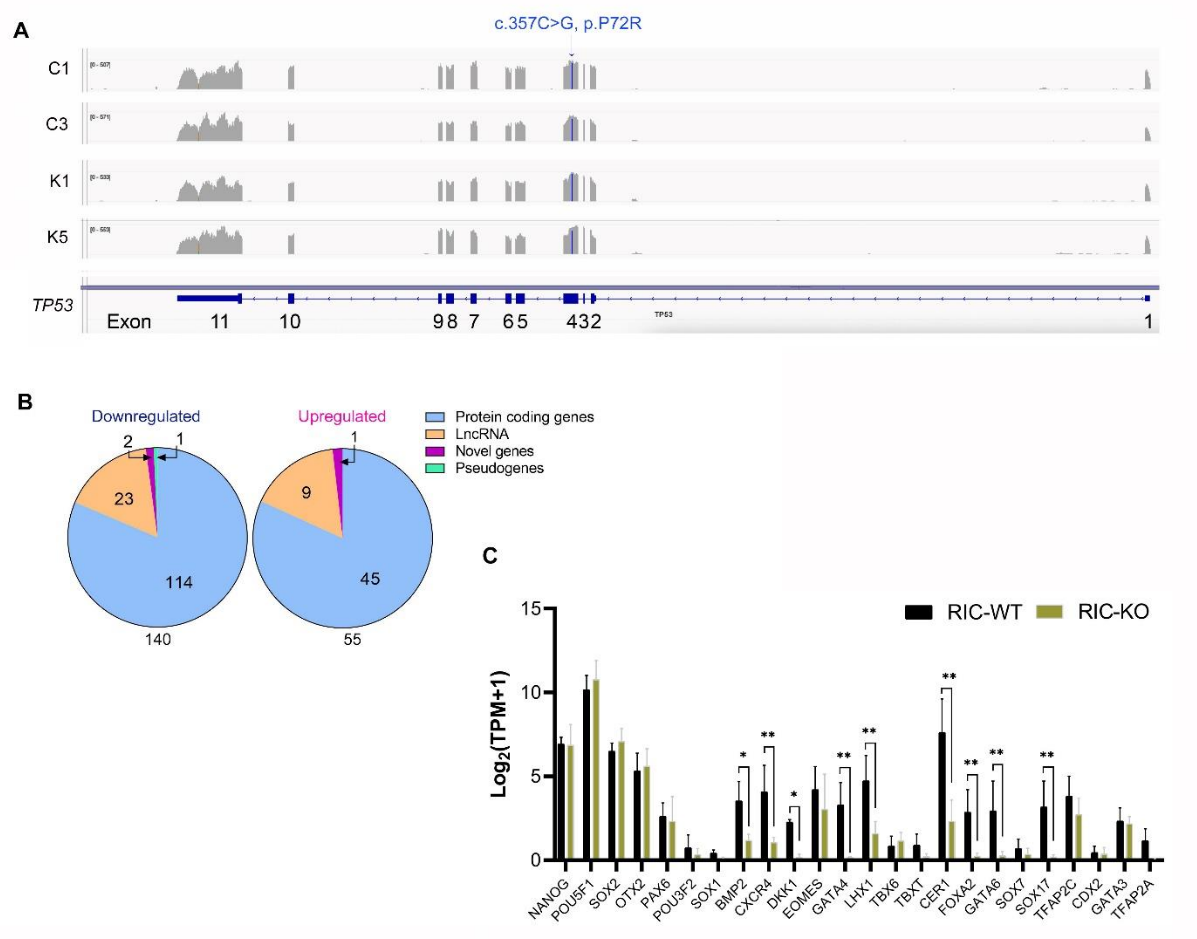

Figure S3. Differentially expressed genes in RIC-KO hESCs by RNA-seq analysis.

(A) Mapping TP53 reads onto the gene. Blue arrow points to a common polymorphism identified in exon 4 in all H1 hESC derivatives.

(B) Pie-chart showing distribution of differentially expressed genes in RIC-KO hESCs.

(C) Bar chart comparing the expression of representative lineage-associated transcription factors between WT and RIC-KO hESCs. Data are presented as mean ± SD (n = 4). * & **, $p < 0.05$ and 0.01, respectively by unpaired two tail t test.

Related to Figure 3

Figure S4

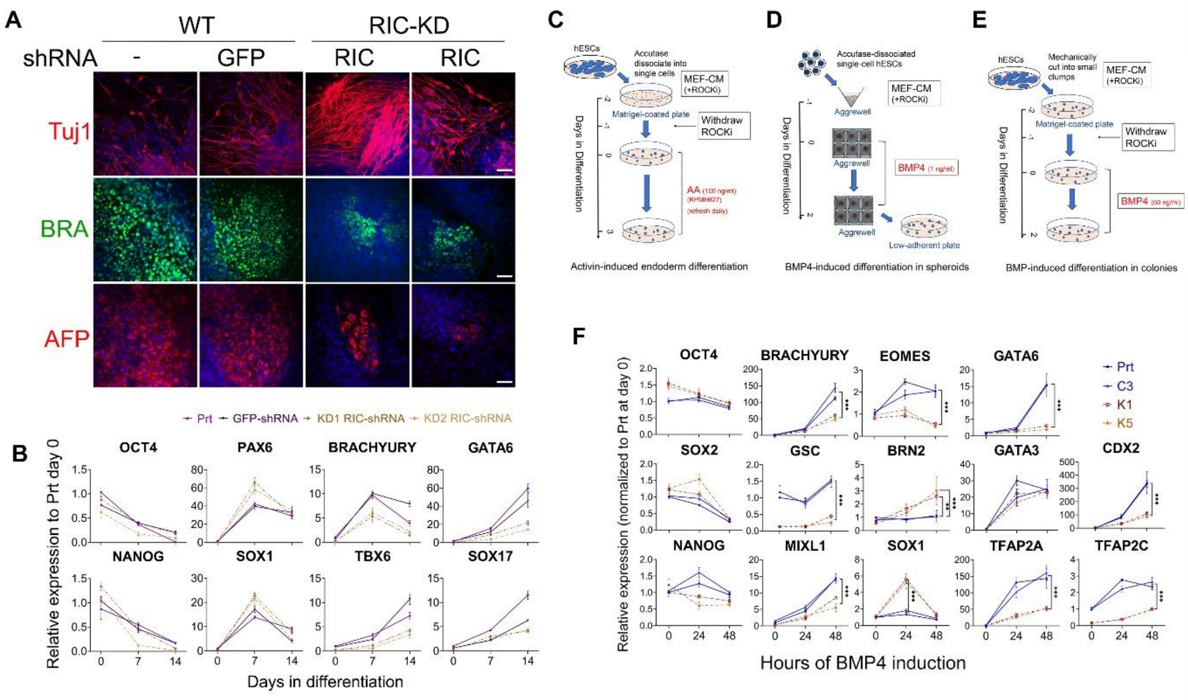

Figure S4. Reduced mesoderm/endoderm differentiation in RIC-KD and RIC-KO hESCs.

(A) Immunostaining of the three germ layer markers in WT and RIC-KD H7 hESCs after 14 days of EB differentiation (n = 2). Scale bar = 50 µm. The RIC-KD images represent two independent KD lines.

(B) Dynamic expression of indicated genes during EB differentiation in WT and RIC-KD H7 hESCs. Data are presented as mean ± SD of 6 measurements of 2 independent differentiation experiments.

(C) Schematic of Activin-induced endoderm differentiation.

(D) Schematic of BMP4-induced differentiation in spheroids.

(E) Schematic of BMP4-induced differentiation in hESC colonies.

(F) Expression of various lineage markers by RT-qPCR in BMP4-induced differentiation in WT and RIC-KO hESCs. Data are presented as mean ± SD (n = 3). ** & ***, *p* <0.005 and 0.0005 by two-way ANOVA.

Related to Figure 4.

Figure S5

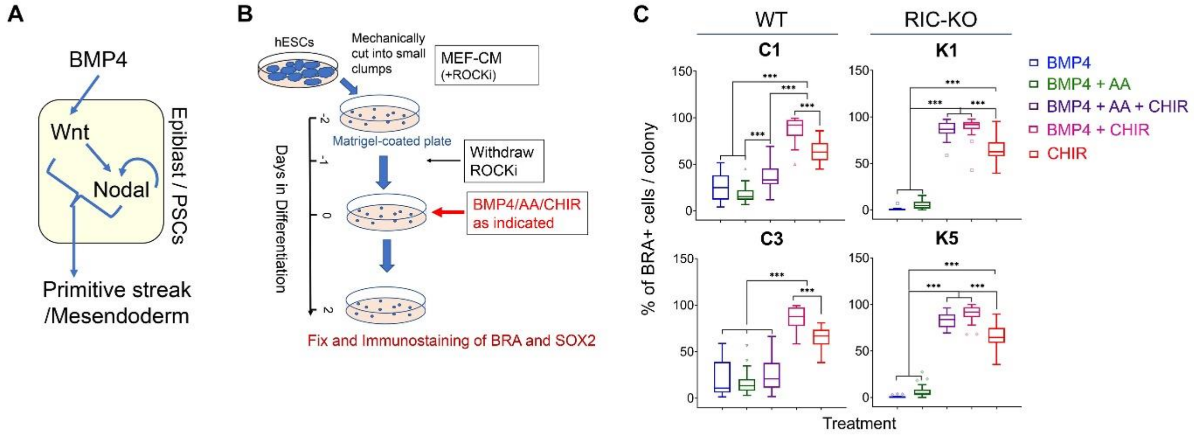

Figure S5. WNT and Activin signaling differentially affect BMP4-induced mesendoderm differentiation in WT and RIC-KO hESCs.

(A) Diagram illustrating signaling pathways involved in the regulation of gastrulation and mesendoderm differentiation.

(B) Schematic of the experimental procedure in the differentiation of hESCs with combined activation of BMP4, WNT and AA (Activin A) pathways.

(C) Comparing the effect of indicated pathways on differentiation of BRA+ cells within the same cell line. Data are presented as the Tukey box plot with number of colonies shown in Figure 4F and Figure 5 (n = 3). *, ** and ***, $p < 0.05$, 0.005 and 0.0005, respectively, by one-way ANOVA.

Related to Figure 5.

Figure S6

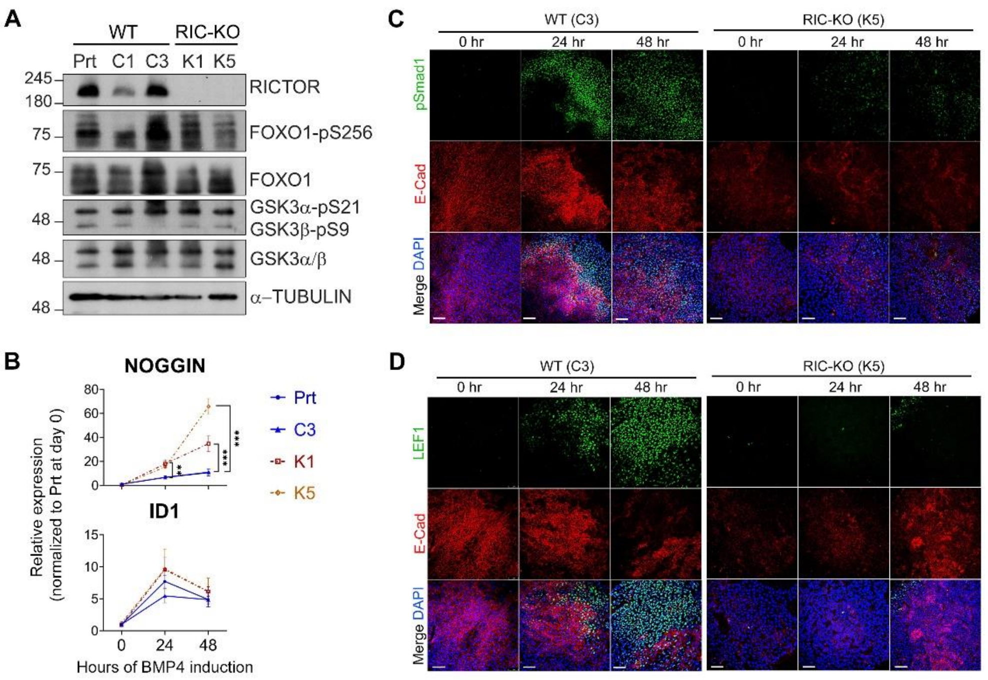

Figure S6. E-CADHERIN modulates BMP4-induced pSMAD1 signal and LEF1 expression.

(A) GSK3 and FOXO1 phosphorylation show similar patterns in WT and RIC-KO hESCs by immunoblotting (n = 2).

(B) Upregulation of BMP4 signaling target genes by RT-qPCR. Data are presented as mean ± SD(n = 3). ** and ***, $p < 0.005$ and 0.0005, respectively by two-way ANOVA.

(C,D) Immunostaining with indicated antibodies in C3 and K5 hESCs as described in Figure 6C,D (n = 3). Scale bar = 100 μm.

Related to Figure 6.

Figure S7

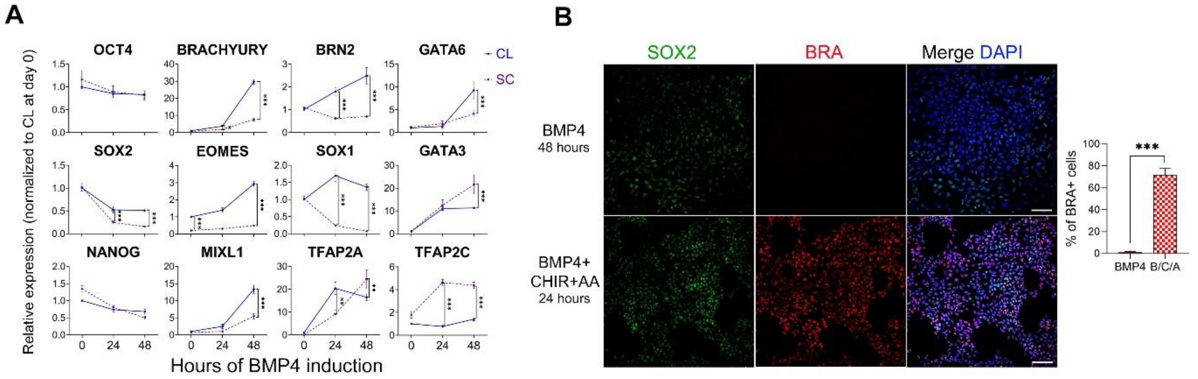

Figure S7. Cell-cell contacts affect BMP4-induced differentiation.

(A) RT-qPCR showing dynamic expression of indicated pluripotent and lineage genes during BMP4-induced differentiation in WT hESCs seeded in colonies (CL) or single cells (SC). Data are presented as mean ± SD (n = 3). ** & *** represent p < 0.005 and 0.0005 respectively by two-way ANOVA

(B) Immunostaining of BRACHYURY (BRA) and SOX2 after differentiation of SC-seeded WT hESCs by indicated treatment. Representative images are shown (left) with quantification of BRA+ cells (right) with data presented as mean ± SD of 3 independent differentiation experiments with at least three random-selected fields being counted for each experiment. ***, p <0.0005 by unpaired t-test. CHIR, CHIR99021; AA, activin A. Scale bar = 100 μm.

Related to Figure 7.

Table S4. List of RT-qPCR Primers used in this study

Gene Name	Forward primer (5'-3')	Reverse primers (5'-3')
AXIN2	GTGAGGTCCACGGAAACTGT	TGGCTGGTGCAAAGACATAG
β-ACTIN	TGTCTGGCGGCACCACCATG	AGGATGGAGCCGCCGATCCA
BRACHYURY	TGCTTCCCTGAGACCCAGTT	GATCACTTCTTTCCTTTGCATCAAG
BRN2	CCCACCCTCCATGGGAGCGT	CCAGCATGCCGTTCACCGTGA
β-CATENIN (CTNNB1)	ATTGTCCACGCTGGATTTTC	TCGAGGACGGTCGGACT
DVL2	TGAGCAACGATGACGCTGTG	GCAGGGTCAATTGGCTGGA
E-CADHERIN (CDH1)	AGCCCTTACTGCCCCCAGAG	GGGAAGATACCGGGGGACAC
EOMES	CGCCACCAAACTGAGATGAT	CACATTGTAGTGGGCAGTGG
FLK1	GGTATTGGCAGTTGGAGGAA	ACATTTGCCGCTTGGATAAC
FOXA2	GGGAGCGGTGAAGATGGA	TCATGTTGCTCACGGAGGAGTA
FZD2	CTTCTCACAGGAGGAGACGC	AAATGATAGGCCGCTCTGGG
FZD7	CGCCTCTGTTCGTCTACCTC	TCATGATGGTGCGGATACGG
GATA3	TAACATCGACGGTCAAGGCA	AGGGATCCATGAAGCAGAGG
GATA6	CAGCAAAAATACTTCCCCCA	ACTTGAGCTCGCTGTTCTCG
ID1	CAGCCAGTCGCCAAGAAT	ACAGACAGCGCACCACCT
LEF1	AATGAGAGCGAATGTCGTTGC	GCTGTCTTTCTTTCCGTGCTA
LRP6	GACTGGGTTGCACGAAATCT	CGGGGTTCCTCTAAGTCCTC
MIXL1	CCGAGTCCAGGATCCAGGTA	CTCTGACGCCGAGACTTGG
NANOG	TGATTTGTGGGCCTGAAGAAAA	GAGGCATCTCAGCAGAAGACA
N-CADHERIN (CDH2)	CAACGGGGACTGCACAGATG	TGTTTGGCCTGGCGTTCTTT
NOGGIN	GCCAGCACTATCTCCACATCCG	AGCAGCGTCTCGTTCAGATCCT
OCT4 (POU5F1)	TCGAGAACCGAGTGAGAGGC	CACACTCGGACCACATCCTTC
PAX6	TCCGTTGGAACTGATGGAGT	GTTGGTATCCGGGGACTTC
RPL22	TCGCTCACCTCCCTTTCTAA	TCACGGTGATCTTGCTCTTG
SOX1	AACACTTGAAGCCCAGATGGA	GCAGGCTGAATTCGGTTCTC
SOX2	GCCGAGTGGAAACTTTTGTCG	GCAGCGTGTACTTATCCTTCTT
SOX17	GGCGCAGCAGAATCCAGA	CCACGACTTGCCCAGCAT
TBX6	AAGTACCAACCCCGCATACA	TAGGCTGTCACGGAGATGAA
TFAP2A	GAGGTCCCGCATGTAGAA	CCGAAGAGGTTGTCCTTGT
TFAP2C	CACCTGTTGCTGCACGATCAGA	AGGAGCGACAATCTTCCAGGGA
WNT3	CGCACGACTATCCTGGAC	GAGGCGCTGTCATACTTGTC
WNT3A	TGTTGGGCCACAGTATTCCT	GGGCATGATCTCCACGTAGT
WNT5A	CCACATGCAGTACATCGGAG	CACTCTCGTAGGAGCCCTTG
WNT8A	TGTGATGGGTCAAACAATGG	TCCTTCCCCTTCTCCAAACT

Table S5. List of antibodies used in this study

Primary antibodies		
**Antigen**	**Supplier, Catalogue #**	**Application and Dilution**
AFP	Sigma, A8452	IF: 1:1,000, IF: 1:500
AKT	Cell Signaling, 9272	IB: 1:1,000
AKT-pS473	Cell Signalling, 4060	IB: 1:1,000
α-TUBULIN	Cell Signalling, 3873	IB: 1:1,000
β-ACTIN	Proteintech, 66009	IB: 1:50,000
β-CATENIN	Cell Signalling, 8480	IB: 1:1,000, IF: 1:100
β-CATENIN	Thermo Fisher, 13-8400	IB: 1:1,000, IF: 1:100
β1-INTEGRIN	Cell Signalling, 9699	IB: 1:1,000
BRACHYURY	Cell Signaling, 81694	IB: 1:1,000, IF: 1:200
β-TUBLIN III (TUJ1)	Bio-Techne, MAB1195	IF: 1:200
E-CADHERIN	Thermo Fisher, 13-1700	IB: 1:1000, IF: 1:200
ERK1/2-pT202/Y204	Cell Signalling, 9106	IB: 1:2000
ERK1/2	Cell Signalling, 9102	IB: 1:1,000
FOXO1-pS256	Cell Signalling, 84192	IB: 1:1,000
FOXO1	Cell Signalling, 2880	IB: 1:1,000, IF: 1:100
GAPDH	Santa Cruz, sc-365062	IB: 1:5,000
GSK3α/β-pS21/9	Cell Signaling, 9331	IB: 1:1,000
GSK3α/β	Santa Cruz, sc-71190	IB: 1:500
LEF1	Cell Signaling, 2230	IB: 1:500, IF: 1:200
mLST8	Thermo Fisher, MA5-14993	IB: 1:1,000
mSIN1	Cell Signalling, 12860	IB 1:1,000
mTOR	Cell Signaling, 2983	IB: 1:500
NANOG	Cell Signaling, 4903	IB: 1:2,000, IF: 1:200
Nestin	Bio-Techne, MAB1259	IF: 1:100
OCT4	Cell Signaling, 75463	IB: 1:2,000, IF: 1:200
p70-S6K	Cell Signaling, 9202	IB: 1:1,000
p70-S6K-pT389	Cell Signaling, 9234	IB: 1:1,000
PKCα/βII-pT638/641	Cell Signaling, 9375	IB: 1:1,000
PKCα	Cell Signaling, 59754	IB: 1:1,000
PKCz-pT	Abcam, ab62372	IB: 1:2,000
PKCz	Thermo Fisher, PA517589	IB: 1:1,000
RAPTOR	Cell Signaling, 2280	IB: 1:1,000
RICTOR	Bethyl Laboratories, A300-459A	IB: 1:2,000
SMAD1/5/9-pS463/465/467	Cell Signaling, 13820	IB: 1:1,000, IF: 1:800
SMAD1	Cell Signaling, 6944	IB: 1:1,000
SMAD2-pS465/467	Cell Signaling, 3108, 18338	IB: 1:1,000
SMAD2/3	Cell Signaling, 8685	IB: 1:1,000
SOX2	Bio-Techne, AF2018	IB: 1:2,000, IF: 1:300
SSEA1	Thermo Fisher, MA1-022	Flow: 1:100
SSEA4	Thermo Fisher, MA1-021	Flow: 1:50-1:100
Tra-1-81	Thermo Fisher, MA1-024	Flow: 1:100
ZO1	Thermo Fisher, 33-9100	IB: 1:2,000, IF: 1:200
**Secondary antibodies**		
Goat IgG-HRP	Santa Cruz, sc-2354	IB: 1:10,000

Mouse IgG (H+L)-HRP	Thermo Fisher, G-21040	IB: 1:5,000-1:20,000
Rabbit IgG (H+L)-HRP	Thermo Fisher, 31462	IB: 1:2,000-1:10,000
Mouse IgG3 Isotype Control	Thermo Fisher, MG300	Flow: 1:100
Mouse IgM Isotype Control	Thermo Fisher, 02-6800	Flow: 1:100
Goat IgG-Alexa Fluor™ 488	Thermo Fisher, A-11055	IF: 1:500
Mouse IgG-Alexa Fluor™ 488	Thermo Fisher, A-11001	IF: 1:500
Mouse IgG-Alexa Fluor™ 568	Thermo Fisher, A-11004	IF: 1:500
Rabbit IgG-Alexa Fluor™ 488	Thermo Fisher, A-11008	IF: 1:500
Rabbit IgG-Alexa Fluor™ 568	Thermo Fisher, A-11011	IF: 1:500
